# Neuroprotective effects of Neurotrophin-3 in MPTP-induced zebrafish Parkinson’s disease model

**DOI:** 10.3389/fphar.2023.1307447

**Published:** 2023-11-28

**Authors:** Noor Azzizah Omar, Jaya Kumar, Seong Lin Teoh

**Affiliations:** ^1^ Department of Anatomy, Faculty of Medicine, Universiti Kebangsaan Malaysia, Cheras, Malaysia; ^2^ Department of Medical Sciences, Faculty of Medicine and Health Sciences, Universiti Sains Islam Malaysia, Bandar Baru Nilai, Malaysia; ^3^ Department of Physiology, Faculty of Medicine, Universiti Kebangsaan Malaysia, Cheras, Malaysia

**Keywords:** neurodegenerative disease, neurotrophin-3, *danio rerio*, neuronal survival, neurotoxin, dopaminergic neuron

## Abstract

**Introduction:** Neurotrophin-3 (NT3) is a neuroprotective growth factor that induces the development, maintenance and survival of neurons. This study aims to localize NT3-expressing cells in the adult zebrafish brain and examine the role of NT3 in a zebrafish Parkinson’s disease (PD) model.

**Methods:** Cellular localization of NT3 in adult zebrafish brains was conducted using *in situ* hybridization. Subsequently, adult zebrafish were injected intraperitoneally with 100 μg/g of 1-methyl-4-phenyl-1,2,3,6-tetrahydropyridine (MPTP) and treated with 400 ng/g body weight of recombinant NT3 (rNT3) via intracranial injection 24 h following MPTP injection. The fish were assessed for neurobehavioral, gene expression, immunohistology, and protein analysis on days 3, 5 and 10 post-MPTP injection.

**Results:** Our findings showed that NT3 was extensively expressed throughout the adult zebrafish brain in neurons. Administration of rNT3 has significantly improved locomotor activity, with upregulation of *th1*, *dat*, *ntf3* and *bdnf* gene expressions compared to MPTP-induced zebrafish. Dopaminergic neurons were also significantly increased in the zebrafish brain following rNT3 treatment. ELISA analysis reported raised GST and decreased caspase-3 levels on day 3 of assessment. The trophic changes of rNT3, however, decline as the assessment day progresses.

**Conclusion:** This study is the first to examine the role of NT3 in the adult zebrafish PD model. NT3 has remarkable trophic effects in the zebrafish PD model. However, further study is needed to examine the dosage requirements and long-term effects of NT3 in PD.

## 1 Introduction

Parkinson’s disease (PD) is a multisystem neurodegenerative disorder characterized by heterogeneous clinical features, including a motor symptoms triad of bradykinesia, rigidity, and tremor with non-motor symptoms of insomnia, constipation, and neuropsychiatric symptoms (Faeiz Syezri Adzmin et al., 2023; Shaibdat et al., 2023). A study conducted in Asia to look for the time trend of PD has found at least a 7.9% increment of PD prevalence annually, with vastly increased prevalence in the 80-year-old group, where there is a 21.3% increment annually (Liu et al., 2016). The essential pathognomonic feature of histopathological analysis of brain tissues in patients with PD is the deposition of Lewy bodies and dopaminergic neuronal degeneration in the substantia nigra pars compacta (Nies et al., 2021). Lewy bodies are abnormal aggregations of proteins located intracytoplasmically in a neuron that are characterized as eosinophilic masses, either single or multiple, spherical or elongated (Wakabayashi et al., 2013). α-synuclein is a monomeric protein vital for neuronal survival and a major component in PD for both hereditary and sporadic cases (Spillantini et al., 1997; Wakabayashi et al., 2013). It has been found that α-synuclein is highly expressed in the presynaptic terminal of the brain and peripheral nervous system, especially within nigral dopaminergic neurons (Toni and Cioni, 2015). It modulates synaptic dopamine (DA) release; however, its dysregulation, mutation, or misfolding will result in it being in its insoluble oligomeric state that tends to fibrillate (Jellinger, 2010). This will result at least in a significant part of Lewy body formation, mitochondrial dysfunction, endoplasmic reticulum stress, oxidative stress, proteasome impairment, and disruption of the plasma membrane, resulting in impairment of synaptic DA release and the death of nigrostriatal neurons (Jellinger, 2010; Hu et al., 2017; Longhena et al., 2017).

It has been shown in studies that PD is involved in multiple and widespread mechanisms, namely, oxidative stress, altered mitochondrial function, altered proteolysis, inflammatory change, and excitotoxic mechanisms (Dexter and Jenner, 2013). This opens the possibility of curing the disease by tackling or reversing its pathogenesis. Currently, PD is managed using several agents that aim to address motor symptoms. However, it has led to many detrimental side effects and a wearing-off phenomenon. One substance that has been looked at as a potential agent is neurotrophin (NT). It is a class of neuroprotective growth factor groups secreted from neurons and glial cells that induce a specific neuronal population’s development, maintenance, and survival (Bhardwaj and Deshmukh, 2017; Omar et al., 2022). Neurotrophin-3 (NT3) is one of the members of the NT group, and its actions are known to be mediated by binding to the tropomyosin receptor kinase family (Trk) and p75 pan neurotrophin factor (p75^NTR^) (Sampaio et al., 2017; Skaper, 2018). Binding to the receptor stimulates autophosphorylation and cascades of events that maintain and manage the nervous system.

*In vivo* PD models treated with a combination of NT3 and neural stem cells (NSC) showed significant improvement (Gu et al., 2009). Further study reported that NT3 enhances survival and neuronal differentiation of NSC into the nigrostriatal pathway (Daviaud et al., 2015). NT3 is also protective in the nigrostriatal pathway by modulating the long-term plasticity of nigrostriatal transmission and restoring long-standing striatal degeneration (Gomez-Pineda et al., 2018). Given its role in neuronal growth, repair, and maintenance, NT3 has been extensively studied for related diseases such as neurodegenerative diseases, neuropsychiatry, brain and spinal injuries.

The utilization of zebrafish as an animal model has witnessed a surge in popularity, attributed to its high reproductive rate, rapid throughput, accessibility, and cost-effective maintenance (Kimmel et al., 1995). Zebrafish has particularly found prominence as a PD model, either through neurotoxin induction or genetic modifications. Neurotoxins that have been previously used, such as 6-hydroxydopamine (6-OHDA), 1-methyl-4-phenyl-1,2,3,6-tetrahydropyridine (MPTP), rotenone and paraquat have been shown to mimic the PD features in zebrafish, especially the neurobehavioral changes and the degeneration of the dopaminergic neuron (Benvenutti et al., 2018; Bashirzade et al., 2022; Hettiarachchi et al., 2022; Kim et al., 2022). While genetic modifications hold promise, the resulting phenotypic variations can be quite diverse (Prabhudesai et al., 2016; Sheng et al., 2018; Van Laar et al., 2020). The use of MPTP has gained widespread use in zebrafish models and has recently undergone an extensive review by our research team to determine optimal dosages and assessment time points for establishing a robust and reproducible PD model. In our prior study, we observed that the most effective PD model in zebrafish involved assessments conducted on day 3 following the MPTP insult. However, it is worth noting that any assessments should be completed within a 10-day window post-insult, as beyond this timeframe, zebrafish exhibited the ability to reverse the neurotoxic effects induced (Omar et al., 2023).

NT are known to have distinctive expressions and locations in zebrafish depending on the development period (Mirescu and Gould, 2013). An embryonic study in the zebrafish model has reported the expression of nerve growth factor*,* NT3 (encoded by *ntf3),* and NT6 (*ntf6)* genes in the zebrafish embryogenic models (Nittoli et al., 2018). To the best of our knowledge, there is no study that has investigated the localization and expression of NT3 in the adult zebrafish brain. It would be useful to look into the expression of NT3 in an adult zebrafish brain as a basis for providing us valuable information regarding its function in the adult central nervous system. Therefore, in this study, we aim to localize the expression of *ntf3* mRNAs in the adult zebrafish brain by *in situ* hybridization (ISH). Furthermore, although there is a vast amount of data reporting the beneficial effects of NT, there is a lack of *in vivo* studies, particularly involving the zebrafish model, on the role of NT3 in PD. Thus, this study also aimed to examine the effect of recombinant human NT3 (rNT3) treatment in a zebrafish 1-methyl-4-phenyl-1,2,3,6-tetrahydropyridine (MPTP)-induced PD model.

## 2 Materials and methods

### 2.1 Animals

A total of 126 adult wild-type zebrafish aged 4–6 months purchased from a local aquarium were housed in 6 L transparent acrylic aquaria at a density of up to 5 fish per liter with the temperature kept at 27°C ± 0.5°C, under a 14:10 h (light:dark) controlled photo regimen. The fish were fed adult zebrafish food (New Life Spectrum, Thera + A, USA) twice daily. The fish were grouped into Control, MPTP and NT3 groups. The control groups received no treatment. The MPTP group was induced by intraperitoneal MPTP injection diluted in normal saline, while the NT3 group received rNT3 injection after intraperitoneal MPTP injection. All experimental procedures were conducted in accordance with ethical approval by the Animal Ethics Committee of Universiti Kebangsaan Malaysia (ANAT/FP/2021/SEONG LIN/24-MAR./1167-MAY-2021-APR.-2024). All of the study protocols can be accessed in Supplementary Table S1, S2.

### 2.2 *In situ* hybridization probe preparation

ISH was conducted to localize the mRNA of the *ntf3* gene in the adult zebrafish brain. The *ntf3* probe was prepared using a 2-step PCR amplification with DIG labeling according to Hua et al. (2018). Briefly, the first PCR uses cDNA extracted, and forward and reverse *ntf3* ISH primers (Table 1), with Taq DNA Polymerase with a Standard Taq Buffer kit (Cat#M0273, New England Biolabs, Massachusetts, USA). The PCR cycle was set with initial denaturation at 95°C for 3 min, followed by 35 cycles of denaturation at 90°C for 30 s, annealing at 60°C for 30 s, and extension at 72°C for 30 s. The final extension was conducted by incubating the sample further at 72°C for 10 min. The PCR products were extracted using 2% gel electrophoresis and purified using Monarch^®^ DNA purification kit (Cat#T3010S, NewEngland Biolabs).

**TABLE 1:** Gene-specific primers used in this study.

Gene	Accession number	Primer sequence (5′- 3′)	
*ntf3*	NM_001327813.1	F	ATT​TCC​TCA​CCG​ATG​CTA​TG
		R	GGT​CCT​GTT​TGT​AAC​CCA​AT
		R EX + T7	CAG​TGA​ATT​GTA​ATA​CGA​CTC​ACT​ATA​GGG​AGA​GGT​CCT​GTT​TGT​AAC​CCA​AT
*th1*	NM_131149.1	F	TGG​ATC​AGG​ATC​ACC​CAG​GA
		R	GTA​GAC​CTC​CCG​CCA​TGT​TC
*th2*	NM_001001829.1	F	GAA​TGC​CAC​ATG​GGA​GGT​TT
		R	AGC​TGA​GGG​ATC​TGG​TCT​TCT
*dat*	NM_131755.1	F	GAG​TCG​GGT​TTG​GTG​TGC​TA
		R	GGC​GTC​TCT​GTA​GCA​GTT​GT
*βact1*	NM_131031.1	F	GCC​TTC​CTT​CCT​GGG​TAT​GG
		R	ATG​TCC​ACG​TCG​CAC​TTC​AT

The purified PCR products were subjected to the second PCR amplification, using a reverse *ntf3* primer extended with a T7 polymerase nucleotide sequence. The second PCR product underwent gel electrophoresis and DNA purification as described previously. The second PCR product was labeled using DIG RNA labeling kit (Cat#11277073910, Roche, Mannheim, Germany), and kept at −80°C before use.

### 2.3 *In situ* hybridization for *ntf3* gene

Adult fish (n = 10) were euthanized using ice water immersion kept at 0°C–2°C, before the dissection of the brain, ensuring all brain parts were included, i.e., the olfactory bulb (OB), telencephalon, optic tectum (TeO), cerebellum and brain stem. Extracted brains were fixed in 4% paraformaldehyde (PFA) for 6 h at 4°C, cryoprotected in a 20% sucrose solution overnight at 4°C and embedded in Optimal Cutting Temperature compound (Cat#4583, Sakura Finetek, Japan). Sagittal (n = 4) and coronal (n = 6) sections (14 µm thickness) were cut with a cryostat and thawed-mounted onto silane-coated glass slides until further processing.

ISH was performed as per Teoh et al. (2015) with modifications. Briefly, sections were permeabilized with 0.2 M HCl/phosphate-buffered solution (PBS) for 10 min, followed by prehybridization at 80°C for 10 min, and hybridized with DIG-labeled riboprobes (4 μg/mL) at 55°C overnight in a humidified chamber. After hybridization, sections were washed and blocked with 10% normal goat serum (Cat#X,090,710, Agilent Dako, California, USA) for 30 min at room temperature. DIG signals were detected with an unconjugated mouse anti-DIG antibody (Cat#AB420, Abcam, Cambridge, United Kingdom, diluted 1:200) overnight, followed by incubation with goat anti-mouse-alkaline phosphatase conjugated (Cat#AB119345, Abcam, diluted 1:1000) for 1 h at room temperature (Nies et al., 2021). Chromogenic development was achieved with 5-bromo-4-chloro-3-indolyl-phosphate/tetranitroblue tetrazolium (Cat#AB7413, Abcam). The sections were visualized under a light microscope, and images were captured using an Digital Slide Scanner (WINMEDIC: Win 20, Shandong, China) and viewed with ZYFViewer version 2.0.1.11 software (WINMEDIC Technology Co. Ltd, Shandong, China).

### 2.4 Double-labelling fluorescence ISH with immunohistochemistry

Cells expressing *ntf3* were characterized by double-labelling with either a marker protein for neurons (HuC/D), astrocytes (glial fibrillary acidic protein, GFAP), or dopaminergic neurons (anti-tyrosine hydroxylase, TH). ISH of *ntf3* was carried out as described above. DIG signals of *ntf3* probes were detected using fluorescein isothiocyanate-conjugated goat anti-mouse (IgG) secondary antibody (Cat#AB6785, Abcam, diluted 1:1000). The DIG-labeled sections were then incubated with either rabbit polyclonal anti-HuC/D (Cat#AB210554, Abcam, diluted 1:500), rabbit polyclonal anti-GFAP (Cat#GTX128741, GeneTex, California, USA, diluted 1:250), or rabbit polyclonal anti-TH (Cat#AB229333, Abcam, diluted 1:500) overnight at 4°C. After primary antibody incubation, the sections were incubated with tetramethylrhodamine-conjugated goat anti-rabbit (IgG) secondary antibody (Cat#AB6718, Abcam, diluted 1:1000) for 1 h at room temperature. The sections were mounted under glass coverslips using Anti-Fade Fluorescence Mounting medium (Cat#AB104135, Abcam). Images were captured using a fluorescence microscope (Eclipse 90i, Nikon, Tokyo, Japan).

### 2.5 PD model induction

The PD model was developed according to the protocol described by Omar et al. (2023). Briefly, a single dose of 100 μg/g of body weight (bw) per fish of MPTP (Cat#M0896, Sigma-Aldrich, St Louis, MO, USA) was administered intraperitoneally using a 30G needle. The fish were assessed on days 3, 5 and 10 post-MPTP injections.

### 2.6 Recombinant NT3 treatment

Following 24 h post-MPTP injection, the fish were anesthetized with 0.0035% benzocaine and were transferred individually to a slender slitted surgical bed in a supine position. A small slit (200 µm) on the skull was made using a 30G barbed-end needle and around 0.5 mm of depth in the cranium above the anterior portion of the optic tectum, gently without damaging the brain (Kizil and Brand, 2011). The tip of the glass capillary was maneuvered through the incision site and was oriented towards the telencephalon without contacting the brain parenchyma. rNT3 solution (Cat#AB9792, Abcam) was injected intracranially using the microinjector (IM-9B, Narishige, Tokyo, Japan) via the glass capillary.

Before initiating the study, a dose-response investigation was carried out since the administration of rNT3 in zebrafish had not been previously examined. Fish (n = 6) were divided into different groups, each administered with doses ranging from 50 ng/g to 1000 ng/g of bw (Demel et al., 2011; Gomez-Pineda et al., 2018; Wei et al., 2018). The survival of the fish was observed for 10 days. The resulting data were plotted versus the probits, and the Lethal Dose 50 (LD_50_) was determined (Randhawa, 2009; Hamidi et al., 2014). Further investigations used doses ranging from 10% to 70% of the LD_50_. The fish were assessed for changes in locomotion, gene expression, and survival for up to 10 days post-injection. The dose selected to be administered in the study was either the lowest dose regimen with positive findings or the highest dose regimen that yielded 100% survival.

### 2.7 Locomotor analysis

Locomotor activity was examined by placing the fish individually in a 2.5 L system water in a water tank. The side view of the fish swimming pattern was recorded using a webcam (C992 pro stream, Logitech) for 5 min. Videos were analyzed using SMART tracking device (Smart 3.0.02, Panlab Harvard Apparatus^®^), for the total swimming distance, swimming speed, time spent on the top half of the tank, and latency to reach the top half of the tank (Cachat et al., 2010).

### 2.8 Gene expression analysis

Fish were euthanized using ice water immersion at 0°C–2°C. The dissected brains were homogenized with 400 µL of TRIzol reagent (Cat#15596026, ThermoFisher, Waltham, MA, USA), and RNA was extracted per the manufacturer’s protocol. A total of 500 ng extracted RNA was reverse transcribed to cDNA with Protoscript^®^ First Strand DNA Synthesis Kit (Cat#E6300S, New England Biolabs). The cDNA was subjected to qPCR for *th1*, *th2*, and *dopamine transporter* (*dat*) for the DA biosynthesis and uptake; *brain-derived neurotrophic factor* (*bdnf*) and *ntf3* for the NT genes. *β-actin1* (*βact1*) was used as a reference gene in this study. The cDNA samples were mixed with the desired primers (Table 1) and Luna^®^ universal qPCR Master Mix (Cat#M3003L, New England Biolabs). The data were analyzed based on the relative expression against the *βact1* gene using the formula 2^−ΔΔCq^.

### 2.9 NT3-expressing cells and dopaminergic neuron immunohistochemistry

Fresh brain tissues were extracted, as mentioned previously. Before staining, the tissue sections were washed with PBS and blocked with peroxidase block. The tissue sections were incubated with mouse anti-TH monoclonal antibody (Cat#22941, Immunostar, USA, diluted 1:500) or mouse anti-NT3 antibody (Cat#GTX83984, GeneTex, California, USA, diluted 1:500), and ARK (Animal Research Kit) peroxidase kit (Cat#K3954, Agilent, Santa Clara, CA, USA) overnight. Tissue sections were treated with Streptavidin-HRP, and DAB + chromogen substrate. Subsequently, sections were cover-slipped, sealed with DPX mountant (Fisher Chemical, USA), and viewed under a light microscope. Immunoreactive cells (TH cells) were analyzed based on overall positive cell groups reported previously (Rink and Wullimann, 2002; Wullimann and Rink, 2002; Filippi et al., 2010; Yamamoto et al., 2010). The regions that were analyzed for the TH cells included the olfactory bulb (OB), subpallidum (SP), pretectum (PR), preoptic region (PO), ventral thalamus (VT), and paraventricular organ (PVO), the periventricular nucleus of posterior tubercle (TPp) of the posterior tuberculum (PT). Inter-rater and intra-rater validation of the microscopic evaluation of the chromogenic staining was conducted by an expert pathologist and an anatomist, where the tissue sections were blinded and positive cells were counted. The results were analyzed to validate the cell count method used. Intra-observer reliability and inter-observer reliability were assessed by Cohen’s Kappa (κ) method, where κ value of 0–0.20 was considered poor; 0.21–0.40: fair; 0.41–0.60: moderate; 0.61–0.80: good and 0.81–1:excellent (McHugh, 2012).

### 2.10 ELISA analysis

The expression of DA, glutathione S transferase (GST), caspase-3 (CASP3), BDNF, and NT3 proteins was assessed using a zebrafish ELISA kit (Cat#ELK9307, ELK9306, ELK9308, ELK9305, ELK9690, ELK Biotechnology, Wuhan, China), according to the manufacturer’s protocol. Briefly, brain samples were homogenized with cold PBS. Homogenates were centrifuged for 5 min at 10,000 bpm at 4°C and a total of 50–100 µL of supernatants were collected and processed in the microplates. Optical density (OD) was read at 450 and 540 nm.

### 2.11 Statistical analysis

Data received was updated in the Statistical Package for the Social Sciences version 20 software (SPSS Inc, USA). A suitable statistical analysis was used based on the outcome of the normality test. Statistical data are presented as the mean ± standard error of mean (SEM) and were analyzed with One-Way Analysis of Variance (ANOVA) and *post hoc* Tukey’s test. Independent-sample T-tests were used for paired comparisons between two specific groups. A *p* < 0.05 was considered statistically significant.

## 3 Results

### 3.1 Localization of *ntf3* mRNA in the brain

**FIGURE 1:**
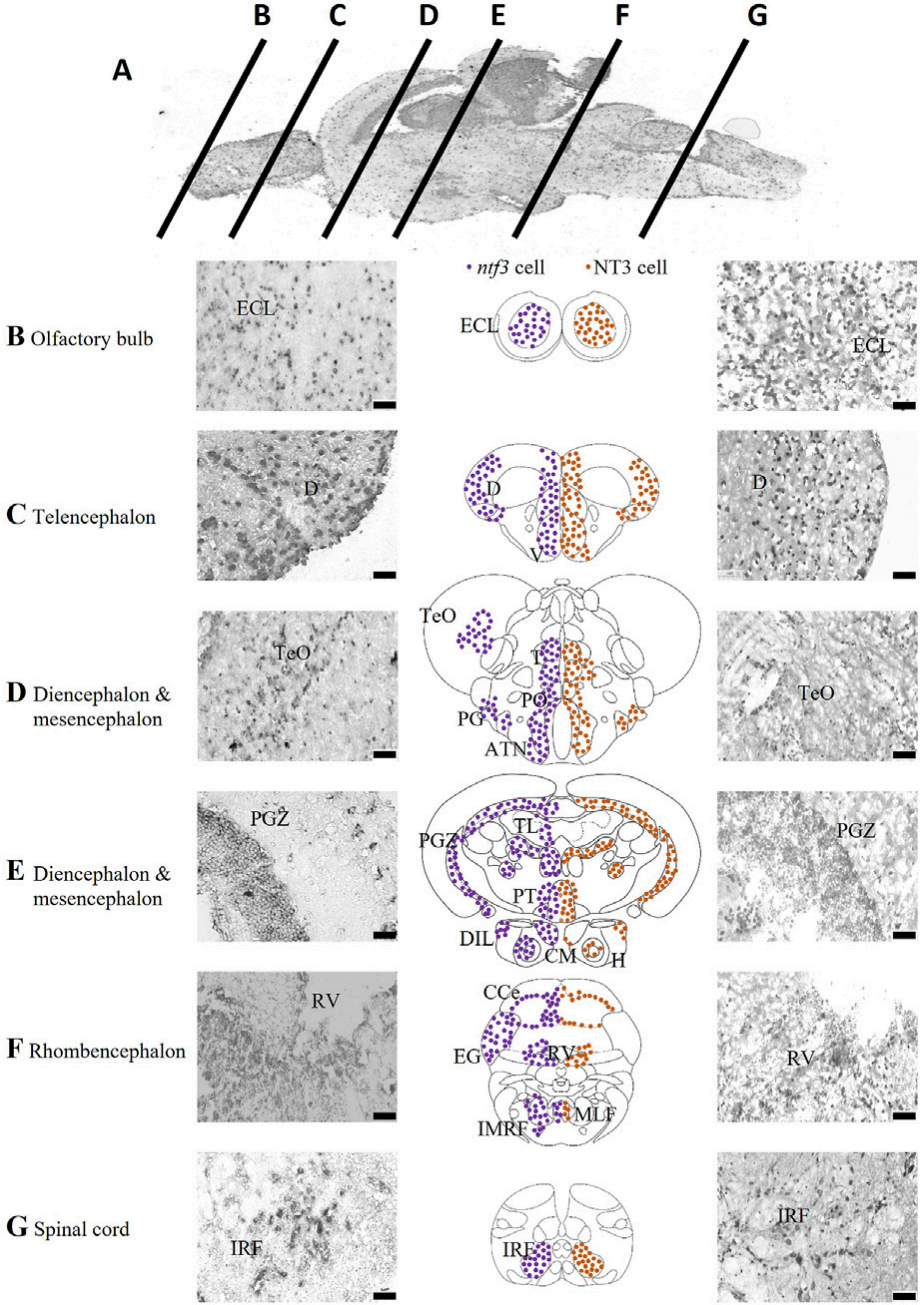
Localization of *ntf3*-and NT3-expressing cells. **(A)** Chromogenic ISH of the adult zebrafish brain with mid-sagittal view. Coronal sections at ×40 magnification of the zebrafish brain from the olfactory bulb **(B)**, telencephalon **(C)**, diencephalon and mesencephalon **(D,E)**, rhombencephalon **(F)**, to the spinal cord **(G)**. Micrographs of the left panels showing *ntf3* cells (ISH), and right panels showing NT3 cells (IHC). Schematic coronal drawings of the brain of zebrafish showing the distribution of *ntf3* cells (purple dots) and NT3 cells (brown dots). Scale bar = 50 μm. ATN: anterior tuberal nucleus; CCe: cerebellum; **(D)** dorsal telencephalic area; DIL: inferior lobe of hypothalamus; ECL: external cellular layer of the olfactory bulb; EG: ementia granularis; H: periventricular area of hypothalamus; IMRF: intermediate reticular formation area; IRF: inferior reticular formation; LLF: lateral longitudinal fascicle; MLF: medial longitudinal fascicle; PGZ: periventricular zone of optic tectum; PO: parvocellular preoptic nucleus; PT: posterior tuberculum; RV: periventricular area of rhombencephalic ventricle; T: thalamic nuclei; TeO: optic tectum; TL: torus longitudinalis V: ventral telencephalic area.

The expression of *ntf3* mRNA was determined with ISH and is summarized in Figure 1. The *ntf3* mRNA-expressing cells (hereafter referred to as *ntf3* cells) have been detected widely in all the sections of the zebrafish brain. In the OB, *ntf3* cells are densely located in the external cellular layer (ECL). In the telencephalon, the distribution is extensive over the dorsal telencephalic area and forms clusters in the ventral telencephalic area, namely, the central nucleus (Vc), dorsal nucleus (Vd), lateral nucleus (Vl), and ventral nucleus (Vv). In the preoptic region, *ntf3* cell clusters are seen in the parvocellular preoptic nucleus (PO), preglomerular nucleus (PG), anterior tuberal nucleus (ATN), and sparsely in the TeO. In the diencephalic and mesencephalic regions, *ntf3* cells are in the thalamic nuclei, posterior tuberculum (PT) of the periventricular nucleus (TPp), paraventricular organ of posterior tuberculum (PVO), periventricular zone of the optic tectum (PGZ), periventricular pretectal nucleus (PR), ATN, posterior tuberal nucleus (PTN), periventricular area of the hypothalamus, corpus mamillarae (CM) and appear sparsely in the diffuse nucleus of the inferior lobe of hypothalamus (DIL). In the caudal part of the diencephalon, dense clusters of *ntf3* cells can be visualized in the torus longitudinalis (TL) and sparsely in the immediate reticular formation area (IMRF). In the rhombencephalon, *ntf3* cells were found in the cerebellum, periventricular area of the rhombencephalic ventricle (RV), intermediate reticular formation (IMRF), and sparsely distributed throughout the ventral segment of the rhombencephalon. *ntf3* cell clusters were found in the inferior reticular formation (IRF) of the spinal cord.

Next, we compared the distributions of *ntf3* cells and NT3-immunoreactive cells (hereafter referred to as NT3 cells, Figure 1). Generally, the distributions of both *ntf3* and NT3 cells were similar. In the OB, NT3 cell clusters were seen in the ECL. In the telencephalon, it was seen in both the dorsal and ventral telencephalic areas. In the dorsal telencephalic area, the central clearing of NT3 cells was more vivid compared to the distribution of *ntf3* cells. In the ventral telencephalic area, the NT3 cells were localized in the Vv, Vl, Vd and Vc, similar to the distribution of *ntf3* cells. In the preoptic region, NT3 cell clusters were seen in the PO and magnocellular preoptic nucleus (PM) with sparing of TeO area that was sparsely positive in ISH. In the diencephalon and mesencephalon, NT3 cell clusters were observed on thalamic nuclei, TPp, PGZ, PR, ATN, PTN and TL. The hypothalamic clusters were not clear compared to the ISH where the periventricular area of the hypothalamus, CM and DIL were not positive. In rhombencephalon, NT3 cells were found in the cerebellum, periventricular area of the RV, and most significantly in the IMRF.

To compare FISH/FIHC results, a double-labelled FIHC was conducted to analyze the coexpression of NT3 protein with the neuronal and glial markers in all brain regions. HuC/D has been shown to have more than 75% overlap consistently in all regions of the brain from the region of the telencephalon, diencephalon, mesencephalon, rhombencephalon and the spinal cord. Around 10% of HuC/D-positive cells did not colocalize with NT3 cells. In contrast, all NT3 cells coexpress the HuC/D marker. In other words, all NT3 cells were from neuronal origin, but not all neurons expressed NT3. The double-labelling of NT3/GFAP showed less than 10% overlap between these two markers in the OB and the ventral telencephalic area. There is around 10%–25% overlap in the diencephalic and mesencephalic areas, such as the TeO and PT regions. In the rhombencephalon and the spinal cord, sparse coexpression was seen where there was less than 10% staining overlap between GFAP and NT3 protein markers. A study of the sections showed that TH cells co-expressed NT3 protein, especially in the diencephalic and rhomobencephalic areas. The TPp and PVO areas in the ventral diencephalon region were shown to have densely stained neuronal cell bodies that co-express the TH and NT3 proteins with 100% overlap. In the telencephalic area, there is less than 50% overlap between cells that express NT3 and TH in the regions of ECL of the olfactory bulb and the SP of the ventral telencephalon. On the other hand, the mesencephalic area has no TH cell clusters and hence was excluded from this study. The rhombencephalon has a 100% overlap between NT3 and TH stains. However, these regions are known to be adrenergic catecholamines rather than dopaminergic neurons (Supplementary Table S3).

### 3.2 Dual fluorescent labelling of *ntf3* mRNA and neuron-/astrocytes-/dopaminergic neuron markers

**FIGURE 2:**
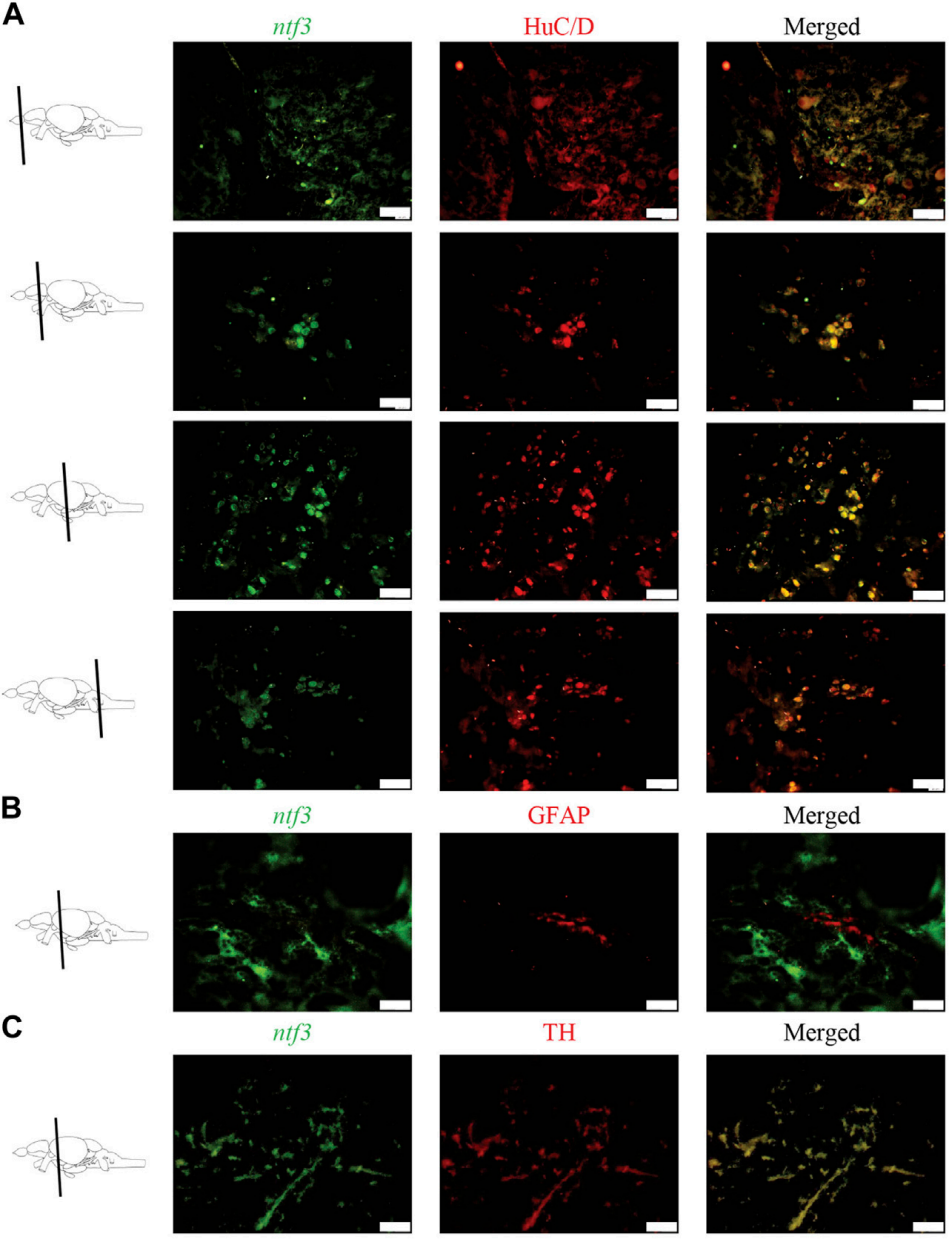
Double labelling fluorescence ISH with immunohistochemistry between *ntf3* mRNA using a DIG-labelled probe and **(A)** neuronal (HuC/D), **(B)** glial (GFAP) and **(C)** catecholamine markers (TH). The co-localization of the neuronal and TH markers with the *ntf3* mRNA was evident. However, this was not true for the glial marker. Scale bar = 50 μm.

Double labeling combining ISH and immunohistochemistry (IHC) was conducted to distinguish which types of cellular markers co-express with the *ntf3* mRNA. Co-expression of *ntf3* cells with HuC/D markers was observed extensively throughout the zebrafish brain, with more than 75% of HuC/D-positive cells co-expressing *ntf3* mRNA (Figure 2A). In contrast, the GFAP marker showed colocalization of less than 10% with *ntf3* cells in all regions of the brain (Figure 2B).

In this study, we also observed that TH cells co-expressed with *ntf3* cells by more than 75% (Figure 2C). Analysis of the sections showed that TH cells co-expressed the *ntf3* mRNA, especially in the diencephalic and rhombencephalic areas. The TPp and PVO areas in the ventral diencephalon region were shown to have densely stained neuronal cell bodies that co-express the TH and *ntf3* mRNA with more than 75% overlap. In the telencephalic area, there is a 50%–75% overlap between cells that express *ntf3* mRNA and TH.

### 3.3 rNT3 dose-response study

**FIGURE 3:**
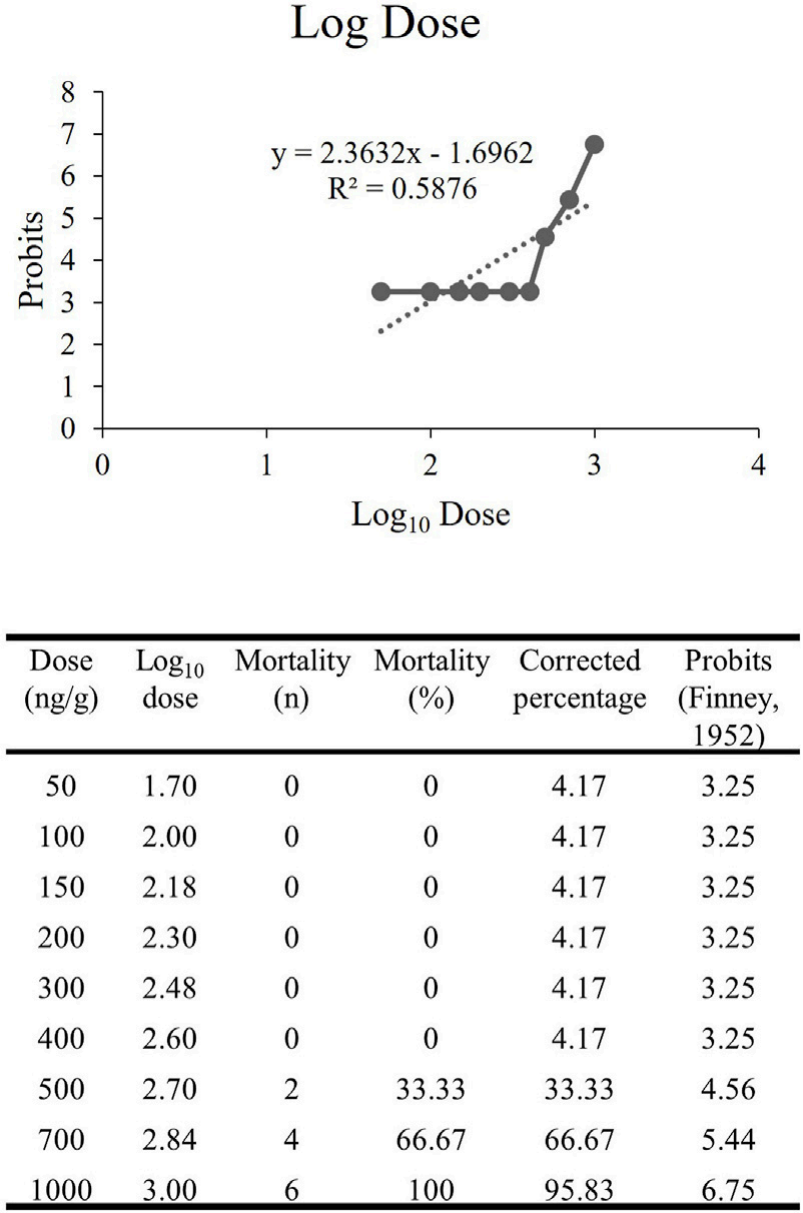
The extrapolated log_10_ dose versus the probits. The probits value is the standardized conversion value from the corrected mortality percentage adapted from Finney’s table (Hamidi et al., 2014).

The dose-response study of rNT3 was conducted with a dose ranging from 50 ng/g to 1000 ng/g (n = 6) in each group. The toxicity effect that caused mortality within 10 days post-injection was calculated and tabulated. The Log_10_ dose was extrapolated (Figure 3) with the probit value. The extrapolated data showed that the Log LD_50_ value is 2.833, converted by antilog to the actual dose of 681.60 ± 1.76 ng/g (Randhawa, 2009). Following the establishment of the LD_50_, further analysis was conducted at doses ranging from 10% to 70% of the LD_50_. Of all the groups, the highest dose with a 100% survival rate and the most significant neurobehavioral and genetic expression improvement was 400 ng/g bw. Hence, this dose was used for all of the procedures afterwards.

### 3.4 Locomotor analysis

**FIGURE 4:**
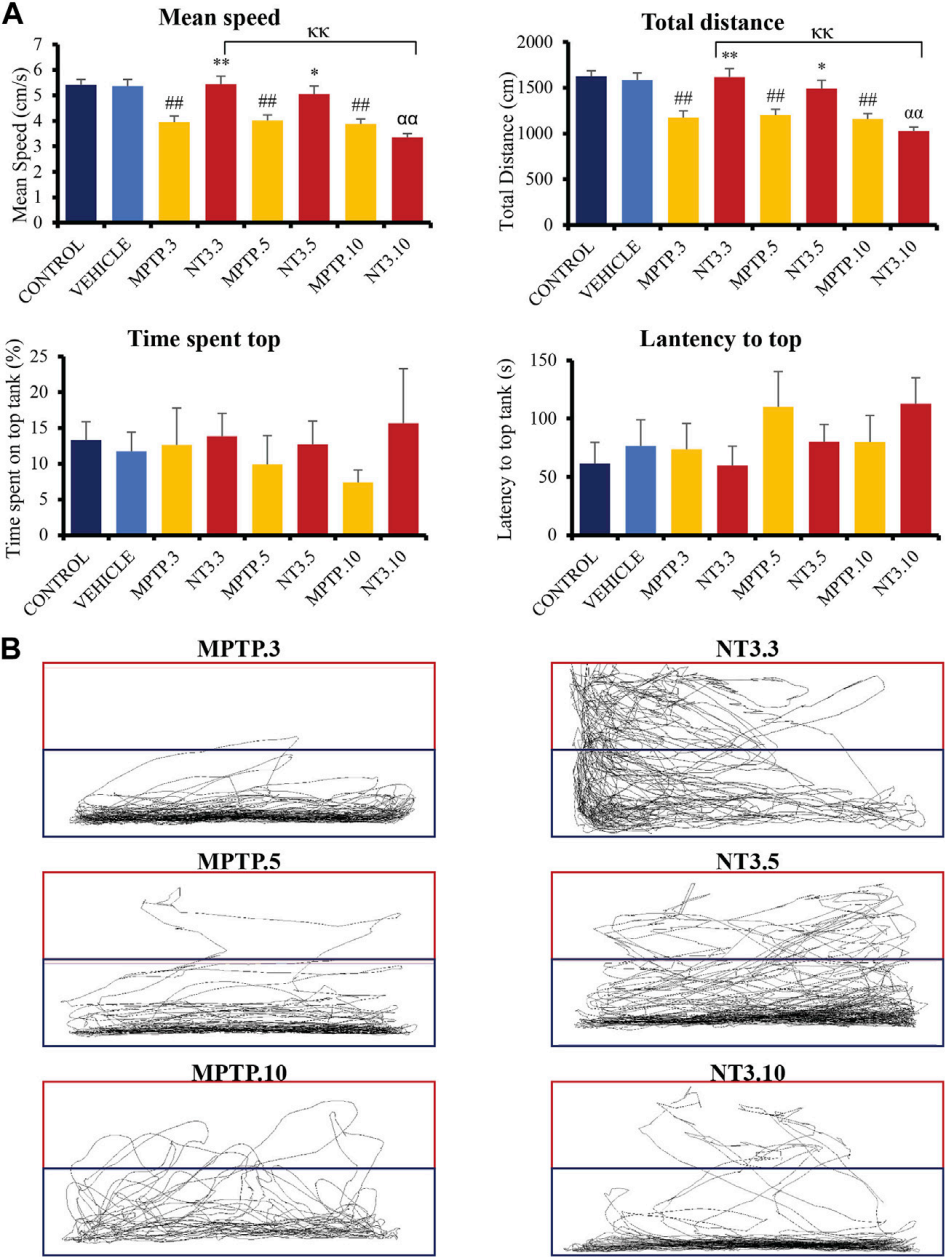
**(A)** Neurobehavioral analysis of the zebrafish group for control, vehicle, MPTP and NT3 treatment groups. The NT3 treatment group significantly improved swimming speed swimming distance, especially in the NT3.3 and NT3.5 groups compared to the corresponding MPTP group. Both NT3 treatment groups were equivocal statistically with the control and the vehicle groups. **(B)** The locomotor tracking between MPTP and NT3 groups where the exploration and the swimming tracts were evidently higher in NT3.3 and NT3.5 group compared to its corresponding MPTP group. However, the changes were not seen in the NT3.10 group. # describes the significant difference between the MPTP and vehicle groups with #*p* < 0.05, ##*p* < 0.01; * describes the statistically significant difference between the NT3 treatment group and its corresponding MPTP group with *p < 0.05, and **p < 0.01; α describes the significant difference between the NT3 group and the vehicle with α *p* < 0.05, αα *p* < 0.01; κ describes the significant difference between NT3.3, NT3.5 and NT3.5 with κ *p* < 0.05 and κκ *p* < 0.01.

The mean swimming speed of the zebrafish PD model was significantly reduced (*p* < 0.001, Figure 4A) following MPTP injection at day 3 (27% reduction), 5 (26% reduction) and 10 (29% reduction) compared to the vehicle group. rNT3 treatment model showed significant improvement in swimming speed at day 3 (5.44 ± 0.32; *p* = 0.001), and day 5 (5.05 ± 0.32; *p* = 0.011), when compared to the corresponding MPTP groups. However, the rNT3-induced improvement in swimming speed plateaued at day 10 (*p* = 0.996).

The total swimming distance corresponds to the mean swimming speed for all groups (Figure 4A). Zebrafish PD model showed a significant decline in the total swimming distance at all time points compared to the vehicle group (*p* < 0.001). rNT3 treatment improved the total distance traveled compared to the MPTP group at day 3 (*p* = 0.001) and day 5 (*p* = 0.011). However, the distance plateaued with no significant improvement at day 10 (*p* = 0.973).

The time lag for the fish to explore the top half of the tank was also analyzed (Figure 4A). Although the latency to reach the top half is reduced in the NT3.3 and NT3.5 groups, it did not reach statistical significance (*p* > 0.05). Similarly, the total time spent on the top half of the tank was also higher in the first two groups. However, it did not reach statistical significance (*p* > 0.05).

In Figure 4B, locomotor tracking is shown comparing the PD model and the rNT3 treatment group. It can be seen that on day 3, especially, a vast and significant increase in locomotor tracking with an increase in upper tank exploration was seen. Nevertheless, as the observation period advances, the distinctions between the PD model group and the NT3-treated group become less pronounced, particularly by day 10 of the assessment.

### 3.5 Gene expression analysis

**FIGURE 5:**
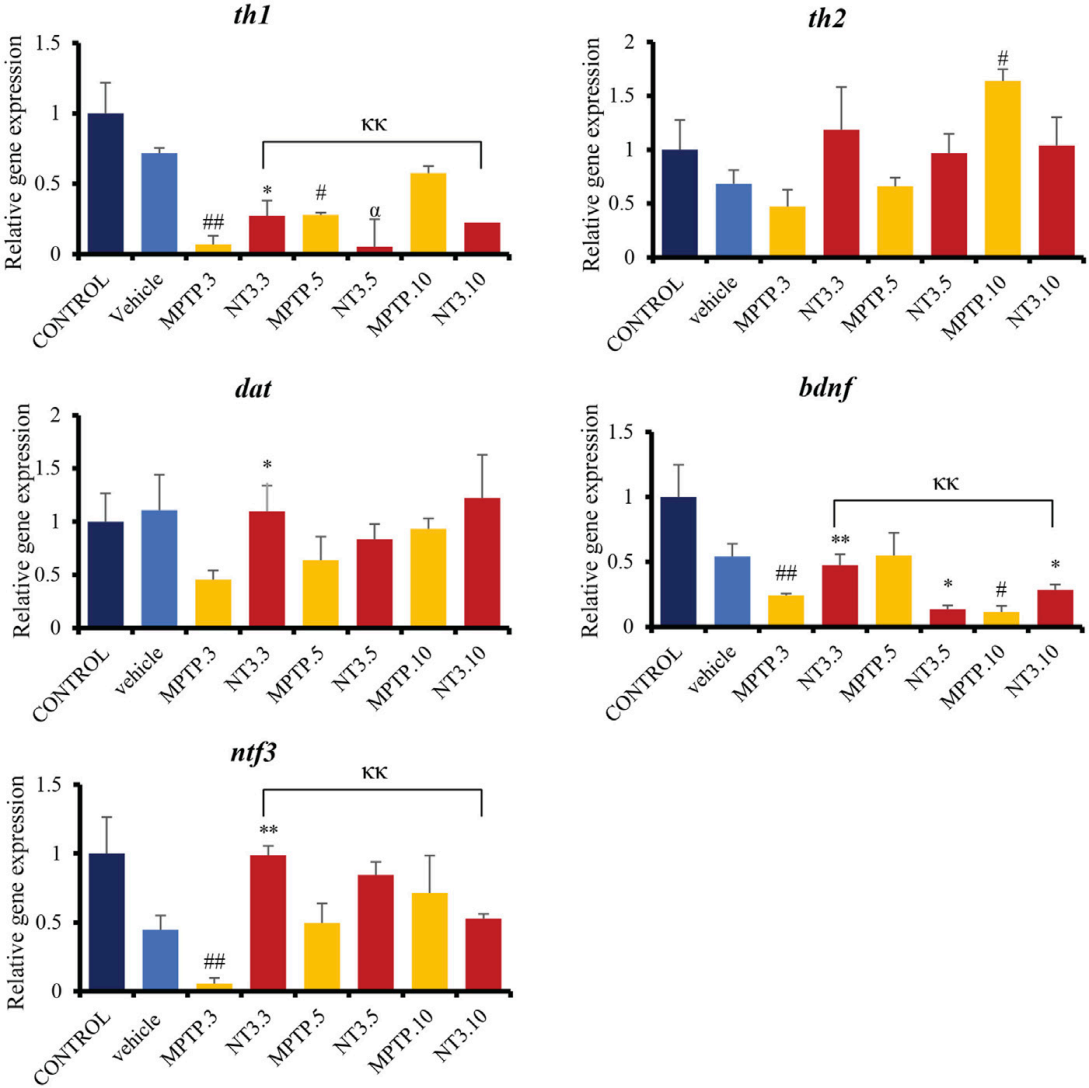
Gene expression between control, vehicle, MPTP and NT3 treatment groups. There was a significant downregulation in *th1* gene expression in MPTP.3, which was reversed with NT3 treatment. *dat* gene has similar pattern with *th2*, where there was a steep increase in expression in *dat* in the MPTP group over 10 days, while the NT3 treatment group had a rather fluctuating pattern. *dat* gene was upregulated in NT3.3 group compared to the corresponding MPTP.3.NT3.3 group has been shown to reverse the downregulation of *bdnf* in the corresponding PD model and become equivocal to the vehicle group. Like *bdnf*, the *ntf3* gene was downregulated in MPTP.3. Following that, a steady increase in gene regulation in MPTP.5 and MPTP.10 became equivocal to the vehicle group and the NT3 groups. # describes the significant difference between the MPTP and vehicle groups with #*p* < 0.05, ##*p* < 0.01; * describes the statistically significant difference between the NT3 treatment group and its corresponding MPTP group with *p < 0.05, and **p < 0.01; α describes the significant difference between the NT3 group and the vehicle with α *p* < 0.05, αα *p* < 0.01; κ describes the significant difference between NT3.3, NT3.5 and NT3.5 with κ *p* < 0.05 and κκ *p* < 0.01.

Gene expression analyses assessed genes related to dopaminergic pathways, namely, *th1, th2*, and *dat*. The assessment was extended by looking at the gene expression of the *ntf3* gene and one of the pioneer’s neurotrophic agents, the *bdnf* gene (Figure 5). The expressions of *th1*, *th2*, *dat, bdnf* and *ntf3* were downregulated in the MPTP group at day 3 and 5 (*p* < 0.01), compared to the vehicle group. On day 10, *th1* gene expression shows no difference between MPTP and vehicle group (*p* = 0.521), while *bdnf* and *ntf3* gene expressions remain significantly downregulated compared to the vehicle group.

Looking at the NT3 group, rNT3 administration upregulated *th1* (*p* = 0.013), *dat* (*p* = 0.022), *ntf3* (*p* < 0.001) and *bdnf* (*p* = 0.009) gene expressions, compared to the MPTP group at day 3. On day 5 and 10, there were no significant differences in *th1*, *dat*, and *ntf3* gene expression between NT and MPTP. The *bdnf* gene expression was significantly downregulated on day 5 (*p* = 0.027), and upregulated (*p* = 0.028) on day 10, compared to the MPTP group.

### 3.6 Immunohistochemistry analysis of TH cells

**FIGURE 6:**
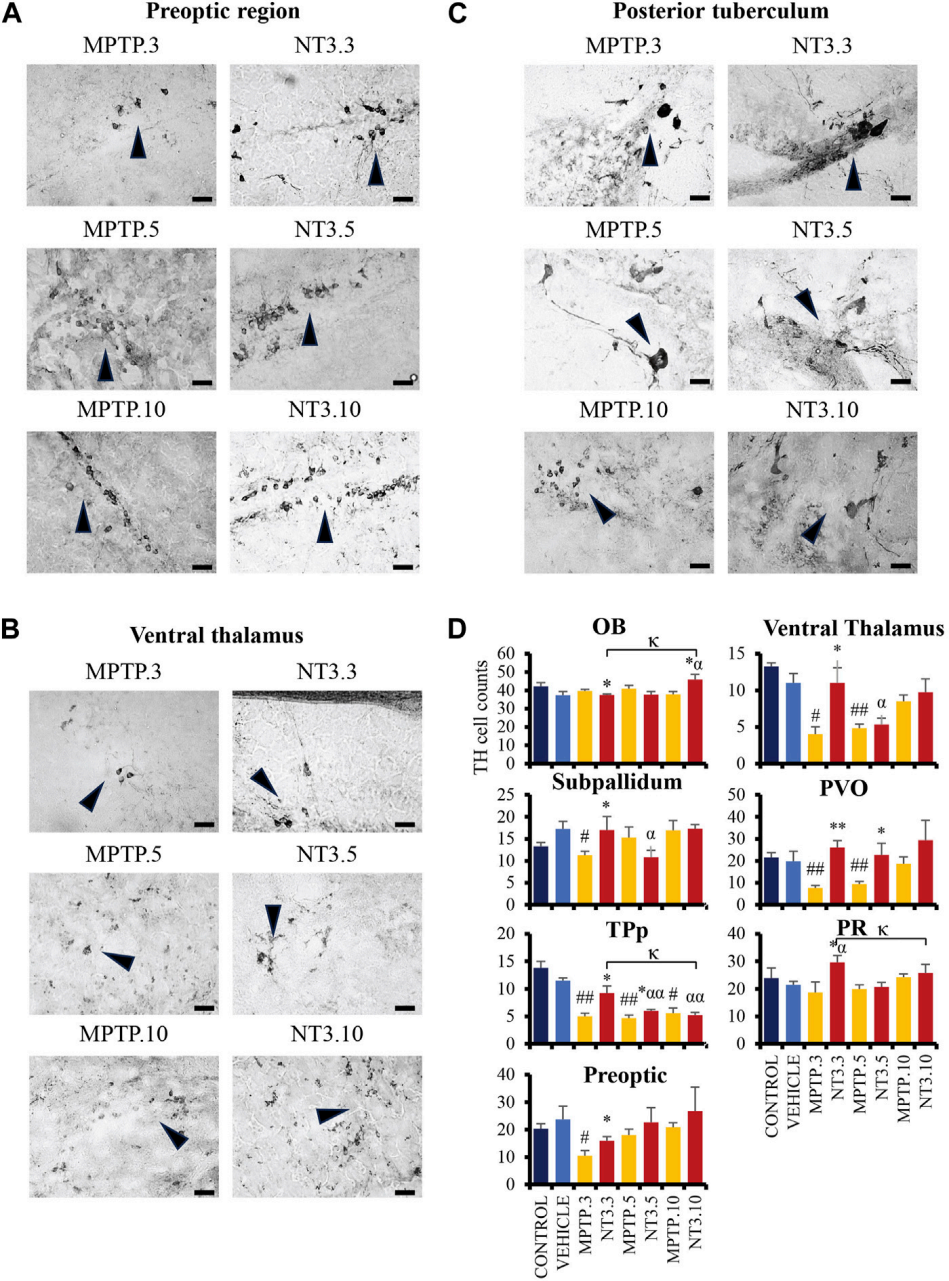
The comparative image of dopaminergic neurons between MPTP and NT3 groups. The TH cell counts in the MPTP group were markedly reduced in the preoptic region **(A)**, ventral thalamus **(B)** and posterior tuberculum **(C)**, compared to the NT3 group. **(D)** TH cell counts in all dopaminergic regions of the zebrafish brain. TH cells were seen to improve in the counts especially in day 3 group. However, becomes equivocal on day 10 of assessment. Arrowheads indicate TH cells. Scale bar = 50 μm. OB: olfactory bulb; PR: periventricular pretectal nucleus; PVO: paraventricular organ of posterior tuberculum; TPp: periventricular nucleus of posterior tuberculum. # describes the significant difference between the MPTP and vehicle groups with #*p* < 0.05, ##*p* < 0.01; * describes the statistically significant difference between the NT3 treatment group and its corresponding MPTP group with *p < 0.05, and **p < 0.01; α describes the significant difference between the NT3 group and the vehicle with α *p* < 0.05, αα *p* < 0.01; κ describes the significant difference between NT3.3, NT3.5 and NT3.5 with κ *p* < 0.05 and κκ *p* < 0.01.

Administration of MPTP reduced TH cell counts in all brain regions examined: PO region (56% reduction, *p* = 0.014, Figures 6A, D), VT region (63% reduction, *p* = 0.010, Figures 6B, D), SP region (35% reduction, *p* = 0.005), TPp region (60% reduction, *p* = 0.004) and PVO regions (61% reduction, *p* = 0.004) after 3 days exposure. On day 5, PD model demonstrated an increase in the TH cell counts: VT region (56% reduction, *p* = 0.002), PO region (23% reduction, *p* = 0.290), TPp region (60% reduction, *p* = 0.001), and PVO region (52% reduction, *p* = 0.009). The increase of TH cells was more apparent at day 10 in the VT region (22% reduction, *p* = 0.176), while the TPp region maintained the cell reduction counts with a 60% reduction (*p* = 0.019). Both the PVO (*p* = 0.843) and PO (*p* = 0.485) regions showed insignificant cell counts compared to the vehicle group at day 10.

Looking into the NT3 group, rNT3 treatment significantly increased TH cell counts in the VT (*p* = 0.039), PR (*p* = 0.026), SP (*p* = 0.025), TPp (*p* = 0.014), and PVO (*p* < 0.001) regions (Figure 6D) at day 3. However, OB showed a significant drop in TH cell count (*p* = 0.011) compared to its corresponding MPTP group. Subsequently, on day 5, TPp (*p* = 0.037) and PVO (*p* = 0.003) showed a persistent increase in TH cell counts in the NT treatment group, compared to the MPTP group. The TH cell counts in other regions were equivocal with the corresponding MPTP group in OB (*p* = 0.311), SP (*p* = 0.222), PO (*p* = 0.408), VT (*p* = 0.616), and PR (*p* = 0.742). At day 10, NT3 group showed no significant difference in TH cell counts in all brain regions when compared with the PD model, except in the OB region which reported a 1.2-fold increase in TH cell counts (*p* = 0.016) compared to its corresponding MPTP group.

### 3.7 ELISA assessment

**FIGURE 7:**
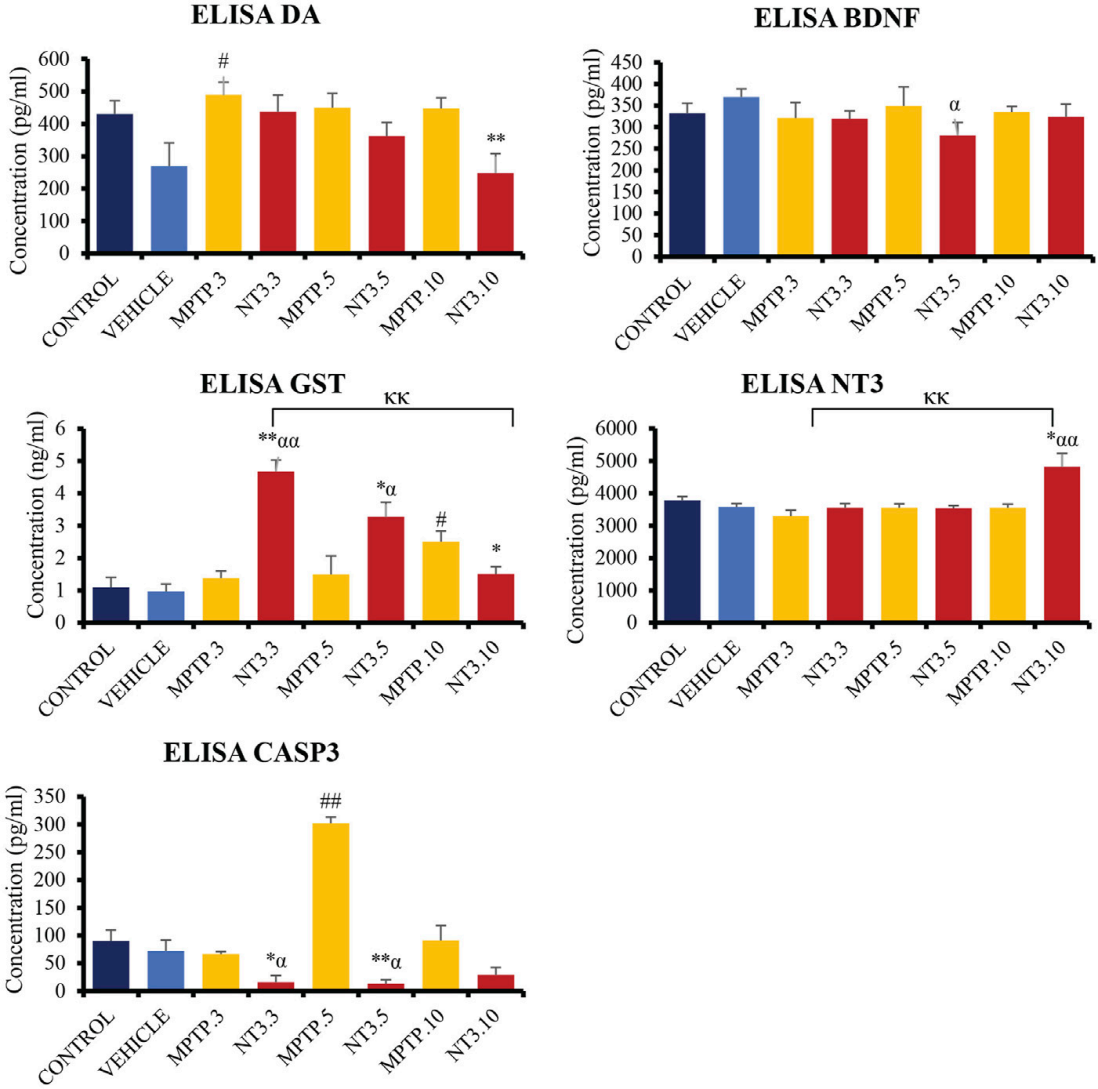
Quantification analysis of DA, CASP3, GST, BDNF and NT3 levels in the zebrafish brain. Interestingly, DA level showed a downward trend in NT3 groups compared to the PD model with the lowest level in day 10 group with highest NT3 level in the similar group. The CASP3 level was significantly lower with a higher GST level in NT3 groups than that in PD group especially in day 3 and day 5 groups. While BDNF was equivocal except for NT3.5 group where the level dropped compared to vehicle group, however equivocal to the corresponding PD group. # describes the significant difference between the MPTP and vehicle groups with #*p* < 0.05, ##*p* < 0.01; * describes the statistically significant difference between the NT3 treatment group and its corresponding MPTP group with *p < 0.05, and **p < 0.01; α describes the significant difference between the NT3 group and the vehicle with α *p* < 0.05, αα *p* < 0.01; κ describes the significant difference between NT3.3, NT3.5 and NT3.5 with κ *p* < 0.05 and κκ *p* < 0.01.

The levels of DA, GST, CASP3, NT3, and BDNF were examined in the zebrafish brain (Figure 7). At day 3, PD model demonstrated a significant increment of DA level (*p* = 0.015), with equivocal CASP3, GST, BDNF and NT3 (*p* > 0.05) when compared with the vehicle group. At day 5, the CASP3 was significantly increased up to 4 folds (*p* = 0.002), compared to the vehicle group, while the DA, GST, BDNF and NT3 levels remained equivocal (*p* > 0.05) to the vehicle group. On day 10, there was a significant increase in the GST level (*p* = 0.012) in the MPTP group compared to the vehicle group. However, other markers such as DA, CASP3, BDNF and NT3 levels showed no significant difference (*p* > 0.05). Despite not showing much difference in the level in the whole brain section, it is worthwhile to reflect on the selective neurodegeneration of the dopaminergic neuron by the MPTP, which may result in equivocal results in the whole brain protein analysis.

Following treatment with rNT3, the protein levels in the zebrafish brain showed some changes. In the NT3.3 group, there was a 4.2-fold reduction in the CASP3 level (*p* = 0.024), and a 3-fold raise in the GST level (*p* < 0.001) compared to its corresponding PD group. The DA, BDNF and NT3 levels remain equivocal (*p* > 0.05). This pattern remains the same on day 5 of assessment, where there is a significant 23-fold reduction in CASP3 level (*p* < 0.001) and a 2-fold increase in GST level (*p* = 0.043) in the rNT3 treatment group compared to the corresponding PD group. There were no changes in the DA, BDNF and NT3 levels between NT3.5 and the corresponding PD model group, the MPTP.5 (*p* > 0.05). The day 10 group showed a peculiar result compared to earlier assessment days. NT3.10 group showed significant reductions in DA (*p* = 0.009); GST (*p* = 0.037) and BDNF levels (*p* = 0.021), with equivocal CASP3 (*p* > 0.05), and an increased NT3 level (*p* = 0.019). Across the assessment days, there is a consistent pattern of decreasing DA and GST levels, with an inclination of CASP3 and NT3 levels.

## 4 Discussion

### 4.1 Distribution of *ntf3* cells in the adult zebrafish brain

We first report the detailed neuroanatomical distribution of *ntf3* and NT3 cells, using ISH and IHC. To our knowledge, this is the first study that has assessed NT3 expression in adult zebrafish brains. The distribution of *ntf3* and NT3 cells was coherent in most of the parts of the adult zebrafish brain, i.e., in the ECL of the OB, in the ventral and dorsal telencephalic areas, in most of the preoptic region, in the diencephalic and mesencephalic areas including the VT, PT, PTN, DIL, and lastly in the IMRF of the rhombencephalon. However, NT3 cells were absent in the TeO and the hypothalamus, where *ntf3* cells appeared abundant. The differences may be due to the ISH technique being more sensitive in detection and probably related to the affinity of the antibody towards the zebrafish antigen in IHC (Yamamoto et al., 2010).

The distribution of the *ntf3*/NT3 cells in the adult zebrafish brain appears to be similar to the region of distribution of *bdnf* mRNA, as described by Cacialli et al. (2016). Some regions have exceptions, such as in the OB where *ntf3* was abundant in the ECL, while *bdnf* mRNA was found more abundantly in the glomerular layer of the OB. On the other hand, *gdnf*, although it has abundant expression in the ECL similar to *ntf3,* but it was sparsely expressed in the PT region, unlike *ntf3* (Wong et al., 2020). Similar to *bdnf*, our study also observed that *ntf3* mRNA was expressed in neurons and not glial cells (Cacialli et al., 2016; Anand and Mondal, 2020), but *gdnf* was more abundantly expressed in glial cells compared to the neuronal marker (Wong et al., 2020). The co-distribution of neurotrophic factors from distinct families with different cellular origins provides compelling evidence supporting the potential multifactorial collaboration in preserving and repairing neuronal physiology.

Pertaining to PD, one striking difference between the zebrafish and mammalian models is the location of the PD locus, which was known historically in the ventral mesencephalon or the midbrain, whereas in the zebrafish, the locations are in the PT of the diencephalon with void TH cell clusters in their mesencephalon (Wullimann and Rink, 2002; Filippi et al., 2010; Yamamoto et al., 2010; Hain et al., 2016). Keeping this concept in mind, our study has shown some coherency where *ntf3/*NT3 were expressed in the PT, similar to a finding in rodent and human brains where *ntf3* mRNA or NT3 proteins were found in their SNpc (Altar et al., 1993; Seroogy et al., 1994). This finding emphasizes the conservation of NT3 function across different species.

### 4.2 Effect of MPTP in adult zebrafish

MPTP is one of the most commonly used neurotoxins to selectively destroy dopaminergic neurons, causing dopamine deficits and manifestations of PD symptoms in various animal models (Najib et al., 2020; Razali et al., 2021). Following intraperitoneal administration of a single dose of MPTP (100 μg/g bw per fish), fish exhibited impairments in motor function as demonstrated by significant reductions in mean speed and total distance traveled. Similarly, previous studies using intraperitoneal injections of 200 μg/g bw, and intracranial injection of 25 mM MPTP in adult zebrafish also demonstrated reduced locomotor activity (Kalyn and Ekker, 2021; Razali et al., 2022).

The current study reported downregulated expression of genes related to dopamine activity (*dat*) and biosynthesis (*th1*) in the MPTP-induced PD model. The selective downregulation of *dat* and *th1* gene expressions and sparing of *th2* expression were in accordance with a previous study that used a paraquat-induced PD model in adult zebrafish (Mohamad Najib et al., 2023). Furthermore, CASP3 which plays a role in the apoptotic signaling pathway, was increased in the zebrafish brain following MPTP exposure. Gene expression of *casp3* was significantly elevated in 96 hours-post-fertilized zebrafish larvae after MPTP exposure (Ren et al., 2022). Similarly, exposure of SH-SY5Y cells to 2 mM MPP^+^ (produced from MPTP by monoamine oxidase B) increased CASP3 activity, suggesting the activation of apoptosis (Chong et al., 2015). Indeed, in the current study, MPTP treatment has been shown to reduce the dopaminergic neuronal population selectively in the PO, VT, TPp and PVO while sparing the OB and SP in the rostral brain region. Similar findings were observed in previous studies that established selective dopaminergic degeneration following MPTP insult (Wen et al., 2008; Caldwell et al., 2019; Lin et al., 2022).

Despite the downregulation of *th1* expression and reduced dopaminergic neurons, our study showed elevated DA level in the zebrafish brain after 3 days post-MPTP injection. In contrast, previous studies reported reduced DA level in MPTP-induced PD models (Anichtchik et al., 2004; Nellore et al., 2013; Sajwan-Khatri and Senthilkumaran, 2023). However, increased DA level was also reported in a paraquat-induced PD model (Bortolotto et al., 2014). The increased DA level could be due to the inhibition of DA re-uptake, as demonstrated by reduced expression of *dat* in this study. It is also possible due to a potential compensatory mechanism to restore the compromised dopaminergic synthesis following MPTP administration.

### 4.3 Effect of rNT3 treatment in MPTP-induced PD model

Since the discovery of NT3, various studies have been conducted utilizing its trophic effect, mainly in the treatment of spinal cord and peripheral nerve injuries (Wan et al., 2014; Wang et al., 2015; Yalvac et al., 2018; Sahenk and Ozes, 2020; Zeng et al., 2022). To the best of our knowledge, this is the first *in vivo* study that was conducted using NT3 as a treatment for brain pathology. A study conducted *in vitro* in a fetal rat brain model has shown that NT3 is vital in neuronal stem cells’ differentiation into neurons and stem cells (Zhu et al., 2012). In our study, intracranial injection of rNT3 into a zebrafish PD model has been shown to significantly improve locomotor activity*, th1* and *dat* genes expression, and increased TH cell counts, with a significant higher GST level and low CASP3 level compared to the PD model following MPTP insult. The tremendous changes were seen on day 3 of treatment following MPTP insult, as most of the markers tested were statistically significant compared to the later days.

Looking at the pattern of the changes, it can be seen that as the day progresses, the speed and swimming distance improvements become less marked compared to the corresponding PD model. There could be a few reasons for this. The first one could be the regenerative capacity of the zebrafish (Kizil et al., 2012), which has been shown to improve any degenerative changes within 12 days following insult (Anichtchik et al., 2004; Vijayanathan et al., 2017). Hence, the trophic changes brought by the NT3 treatment looked less evident as the days went by. Another reason could be due to the short half-life of NT3 itself following *in vivo* injection and its ability to sustain the trophic effects after injection (Poduslo and Curran, 1996; Pradat et al., 2002). Here, we postulate the need for multiple injections. The question remains whether it could repair any damage permanently or if there is any need for multiple dosages following an insult. In another aspect, the anxiolytic property of the NT3 (de Miranda et al., 2020) in zebrafish could be the reason for the locomotor changes within 10 days of treatment. There is a drop in speed, distance, and latency to the top, and an increase in time spent on the top tank could be a change seen in the anxiety model as previously described (Egan et al., 2009).

The level of DA remains equivocal on days 3 and 5 of treatment. However, the DA levels were also not statistically different in the NT3 treatment group compared to the MPTP and vehicle groups. This could be due to MPTP’s selective degeneration of dopaminergic clusters, making the overall level equivocal compared to the control (Lin et al., 2022). Curiously, the DA level dropped significantly in the day 10 of the NT3 treatment group, with a significantly raised NT3 level on the same day. There could be another possibility that external administration of NT3 could acutely promote trophic changes in the area that is damaged but may cause the overexpression of NT3 could lead to a secondary injury (Lee et al., 2016). This was evident in our data, where there is a high peak of overexpression of *ntf3* on day 3, followed by a declining DA level, an increase in NT3 level, a drop in GST, and an increase in CASP3 level on day 10. Although there is no data in the previous study that has tested the oxidative markers in overexpressing NT3-treated animals, most of the studies involving NT3 were mainly *in vitro* or locally treated injuries that had remarkable positive findings on the local effects of NT3.

It is perplexing to acknowledge the unexpected drop in overall DA level with a concurrent increment in NT3 level at day 10 of assessment in the whole brain that was seen in the ELISA results. These findings probe us to understand the importance of cellular equilibrium and the physiological threshold of rNT3 actions. A study looked at the role of NT3 in neuronal homeostasis. This study found that NT3 directly activates the PI3K/Akt pathway and indirectly activates the MAPK and PLC pathways. The former pathway was known to promote neuronal survival and growth of dopamine neuron, inhibiting apoptosis (Long et al., 2021). Still, it also releases other secondary factors that led to the activation of the latter pathways, which resulted in a reduction in neuronal precursor proliferation and inhibited neuronal maturation. They have also found that this inhibition is dose-dependent (Simpson et al., 2003). The PI3K/Akt pathway modifies downstream molecular targets, i.e., GSK-3, mTOR and FoxO3a, to affect oxidative stress, and studies have reported reduced mTOR activity could result in neurodegeneration (Lan et al., 2017; Subramanian et al., 2022). Keeler et al. (2017) has observed that binding of NT3 to TrkA activates this latter pathway that prevents neuronal branching which is a critical step in ensuring neuronal connectivity (Gibson and Ma, 2011). The administration of exogenous NT3 probably prematurely induced the inhibitory pathway that led to these results. In our study, we explored the safe administration of rNT3 in the zebrafish model. However, it would be beneficial to investigate an optimum dose that may or may not need multiple small doses to induce neuronal healing while chronically minimizing its negative feedback mechanism.

The trophic effects of rNT3 were evident in the IHC study of TH cells, where there was a significant improvement in TH cell counts in the treatment group compared to the PD model. This finding further supports the *in vitro* study findings that looked at NT3 as a potential tool for regenerating dopaminergic neurons (Daviaud et al., 2015; Moradian et al., 2017). It has been found that NT3 enhances healing and recovery by regulating neuroinflammation response and neuronal survival and increasing NSC numbers (Huang et al., 2023). The effects of NT3 treatment have greatly improved locomotor behavior in zebrafish, which coherently improved the TH cell counts in the VT, TPp and PVO regions. NT3 treatment showed improvement in the TH cell counts of the PO region; however, it did not reach statistical significance. The changes in TH cell counts in OB and PR were also affected in the NT3 treatment group. This is a rather peculiar finding, especially in OB, where there is a drop in the TH cell count on day 5, which then increased again on day 10. This could be due to glial activation preceding neuronal recovery as reported in a previous study (Azbazdar et al., 2023). The study found that following an insult that caused neuronal damage, glial activation occurred before neuronal recovery. In contrast, the PR region has increased TH cell count specifically in the day 3 group, then returned to baseline and was indifferent to its corresponding PD model group. This could be due to the activation of an orphan nuclear receptor, NR4A2, in the neuronal progenitors in the pretectum area that has been found to play a vital role in triggering the differentiation of dopaminergic neurons (Blin et al., 2008; Chen et al., 2013).

Treatment of rNT3 in the zebrafish PD model has shown remarkable potential in improving locomotor behavior, gene expression, dopaminergic neuronal survival, and prevention of oxidative stress by MPTP, especially during the early days post-treatment. Because this is the first data on the use of the rNT3 *in vivo* model, there is a need to explore a few aspects. Some points need to be addressed for further studies relating to dosage frequency and duration of treatment. The mechanism of why there is a declining improvement in PD features should be explored further, whether it is the dosing frequency issue or the possibility of secondary injury secondary to overexpression. If it is later, the mechanism of injury and the dosing threshold need to be explored. A lower dosage with repeated doses at some intervals may prevent the declining PD features from improving while preventing secondary injuries. If this is the case, the risk of injury during multiple intracranial injection versus no treatment could be raised. Hence, the role of peripheral administration of rNT3 with a carrier that could cross the blood-brain barrier could also be explored.

Further animal studies need to be conducted to examine the long-term effects of rNT3 treatment in PD animal models. From our research, the zebrafish model was able to reverse the MPTP effects from day 10 onward and can be seen to return to the control level by day 30 of treatment. Hence, this could be less suitable for a study that understands the long-term effects of rNT3. However, this model has shown that MPTP does, in fact, cause selective degeneration of dopaminergic neurons, NT3 levels were affected by this induction, and rNT3 treatment does help in recovering the dopaminergic neuron during the peak of PD features in the zebrafish model. Apart from that, further study on how NT3 promotes neuronal healing and regeneration should be explored to understand the feedback mechanism for the treatment protocol for PD.

## 5 Limitation of study

While this study contributes valuable insights into the expression of NT3 and its neuroprotective role in the MPTP-induced zebrafish PD model, it is important to acknowledge certain limitations that may have influenced the outcomes. In this study, we examined the NT3-expressing cells in the zebrafish brain using a mouse anti-NT3 antibody, which has not been used in the zebrafish brain before. The antibody was produced using a human recombinant protein fragment corresponding to amino acids 139–257 of human NT3 (NP_002518) as an immunogen. We did not perform antibody specificity validation, which may have impacted the comprehensiveness of our study. Nevertheless, the zebrafish NT3 amino acid sequence showed 77% similarity when compared to the human NT3 fragment 139–257. Additionally, the evaluation of NT3 effects on the PD model in zebrafish was constrained by the inherent regenerative capacity of the zebrafish brain. As a result, we were unable to conduct assessments beyond day 10, which precluded the examination of the long-term effects of NT3 treatment on PD in this model.

## 6 Conclusion

This study has provided compelling evidence of NT3 localization within the adult zebrafish brain, notably concentrated in the ventricular regions and posterior tuberculum area. This finding suggests its potential involvement in facilitating regenerative neuronal repair, particularly in the context of PD. The administration of recombinant NT3 in the PD model yielded a noteworthy improvement, particularly evident on day 3 of the experiment, highlighting its trophic effects. However, further investigations are warranted to delve into the long-term implications of NT3 and its pharmacodynamic properties as a potential therapeutic intervention for PD.

## Figures and Tables

**Table:** 

**Akt**	Protein kinase B
**ATN**	Anterior tuberal nucleus
**BDNF**	Brain-derived neurotrophic factor
**CASP3**	Caspase-3
**CCe**	Cerebellum
**CM**	Corpus mamillarae
**D**	Dorsal telencephalic area
**DA**	Dopamine
**DIL**	Diffuse nucleus of the inferior lobe of hypothalamus
**ECL**	External cellular layer
**EG**	Ementia granularis
**GFAP**	Glial fibrillary protein
**GST**	Glutathione S transferase
**H**	Periventricular area of hypothalamus
**HuC/D**	Hu protein C/D
**IMRF**	Intermediate reticular formation
**IRF**	Inferior reticular formation
**LD**_**50**_	Lethal dose 50
**LLF**	Lateral longitudinal fascicle
**MAPK**	Mitogen-activated protein kinase
**MLF**	Medial longitudinal fascicle
**MPTP**	1-methyl-4-phenyl-1,2,3,6-tetrahydropyridine
**NGF**	Nerve growth factor
**NSC**	Neural stem cell
**NT**	Neurotrophin
**OB**	Olfactory bulb
**P13K**	Phosphatidylinositol 3-kinase
**P75** ^**NTR**^	p75 pan neurotrophin factor
**PFA**	Paraformaldehyde
**PG**	Preglomerular nucleus
**PGZ**	Periventricular zone of the optic tectum
**PLC**	Phospholipase C
**PM**	Magnocellular nucleus of preoptic region
**PO**	Parvocellular nucleus of preoptic region
**PR**	Periventricular pretectal nucleus
**PT**	Posterior tuberculum
**PTN**	Posterior tuberal nucleus
**PVO**	Paraventricular organ of posterior tuberculum
**RV**	Periventricular area of the rhombencephalic ventricle
**TeO**	Optic tectum
**TH**	Tryrosine hydroxylase
**TL**	Torus longitudinalis
**TPp**	Periventricular nucleus of posterior tuberculum
**Trk**	Tropomysin receptor kinase family
**Vc**	Central nucleus of ventral thalamus
**Vd**	Dorsal nucleus of ventral thalamus
**Vl**	Lateral nucleus of ventral thalamus
**VT**	Ventral thalamus
**Vv**	Ventral nucleus of ventral thalamus

## Data Availability

The raw data supporting the conclusion of this article will be made available by the authors, without undue reservation.
