# Supplementary material for: Neuroprotective effects of Neurotrophin-3 in MPTP-induced zebrafish Parkinson’s disease model
Source: Front Pharmacol. 2023 Nov 28;14:1307447. doi: 10.3389/fphar.2023.1307447 (PMC10713795; doi:10.3389/fphar.2023.1307447)
Supplement: Supplementary file 1 [file DataSheet2.PDF]

<u>Supplementary 2: DNA sequencing for *ntf3* probe</u>

The primers designed in the primer-BLAST database were crosschecked across the non-redundancy database (**Figure 1**). Based on the cross-check, theoretically, the primers are expected to target the *ntf3* gene of zebrafish. Following this, the quality of DNA was amplified by this method by running through the DNA in an electrophoresis machine to check whether the size of the PCR product and the estimated amplicon size tally. The PCR product from the second primer set consistently shows a similar bp length during the first PCR cycle. This DNA product was purified and proceeded with the second PCR cycle (**Figure 2**).

In the second PCR cycle, DNA products were run in the electrophoresis to estimate the DNA size in relation to the DNA ladder. The second PCR product was expected to be more prominent and have a thicker band than the first. In our study, the second PCR product has a wider band (higher DNA concentration) and a slightly bigger size than the first (**Figure 2**). The second PCR product showed consistent base pair size for all tested samples. This PCR product was then purified, and the concentrations were counted using a nano spectrometer. Following this assessment, the PCR products were further assessed on their sequence using the Sanger sequencing method. The PCR product of the second primer set was tested, and the data were cross-checked with the nucleotide-BLAST database. Upon crosschecking the nucleotide sequence, the PCR product was shown to have a 513bp amplicon size with a 99% match with zebrafish *ntf3* mRNA with one codon mismatched (forward) and three mismatched in the reverse primer (**Figure 3** and **4**).

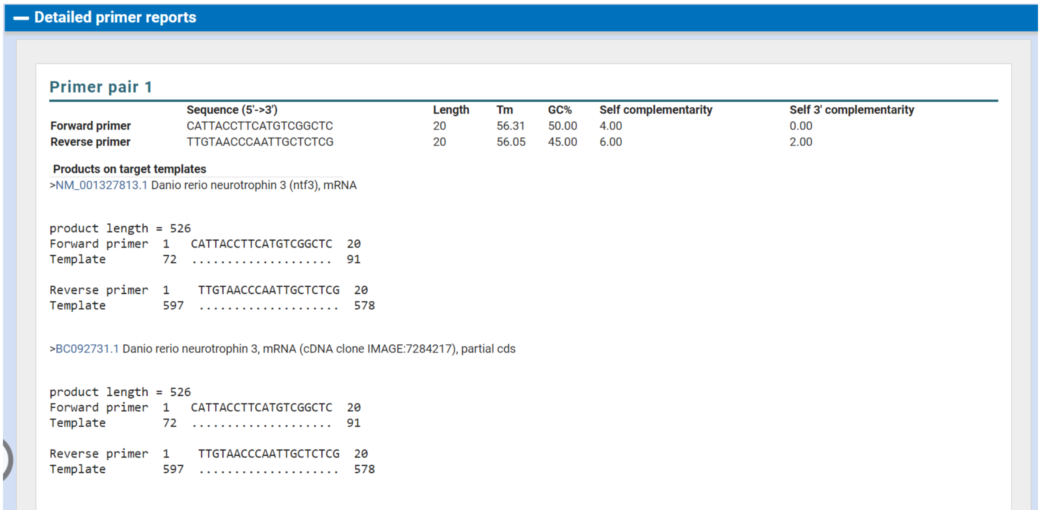

(a)

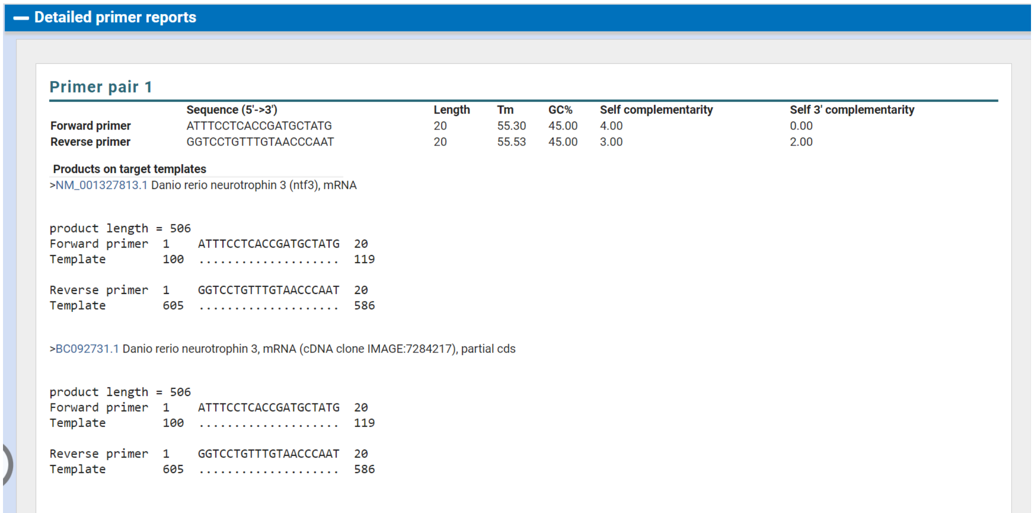

(b)

Figure 1. The target gene using the custom-made primer from the first set (a) and the second set (b). Based on the database, both designs were specific to the *ntf3* gene of zebrafish.

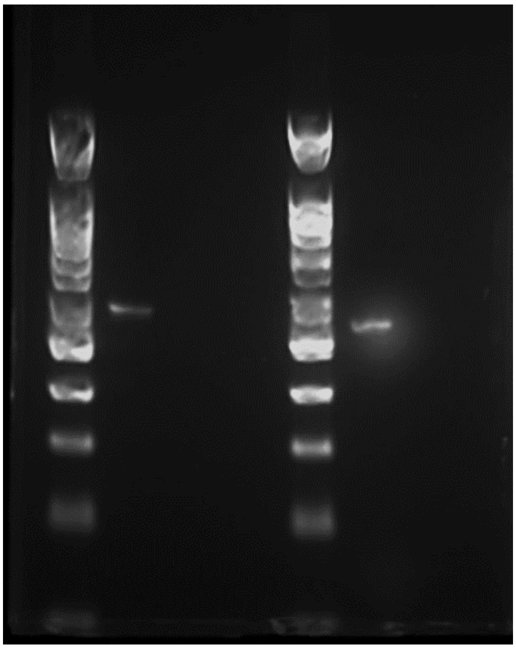

Figure 2. DNA band for the first PCR product (left) and second PCR product (right) using the second set of primers. The DNA ladder was 100 bp, identifying 100 to 1000 bp amplicon size.

```
••• GTGAGACTCTCAGCACGCACCGCGCTTTTCTATCTGGAAACTTACGACAAAAGGATTCGTTATAAACTCCATTACCTTCATGTCGGCT    90
    CTGCTGAATATTTCCTCACCGATGCTATGGTTACCTTTATTACGATCTTACAGGTGAATCTAGTGATGTCCATCTTGCTGTACGTGATGT   180
    TCCTCGCGTACCTTTATGGTATCTCCGCCTCTACAATGGACAAGCAGCGGCCCATGCAGGATCCCATCAGTGCGCTCATCATAAAGCTCC   270
    TGCAGGCTGATATCAGCCGCCAGAAACAGGGCGACGTCATTCCCTCCTCCACCTTAAATGTGTCCACAGTCGCAGACTACAAGGAAAAAG   360
    ACAACAATACCCCCATGGTGGACAGTTCGGAGGAGCTGCTACGCCAGTACAAGCGCTACAACTCTCCACGTGTCCTCTTAAGCGACCGAC   450
    CTCCAATGCAACCACCGGCTTTCTACCTCATGGATAAATTCATCAGCGGTCAGGACTCCAACAAGACGCGGCAGAAACGCTTTGCCGAGC   540
    ACAAGAGCTACAGAGGAGAGTTTTCAGTGTGCGACAGCGAGAGCAATTGGGTTACAAACAGGACCTCAGCGGTGGATTCTCGTGGAAATC   630
    ATGTCACCGTTTTGAATTCCTTCACTGTAAAACAGAAATACACGCAGTACTTTTATGAGACCAAGTGTAAAACTCCTCACTCCCACCGCA   720
    AGAGCCGATGTTTAGGTATTGATGAGAAGCACTGGAATTCGCAGTGCAAAACCACGCAGACTTACGTACGAGCGCTTACTCAGCACAAAT   810
    CGCTGGTTTCTTGGAATTGGATACGGATAGACACGGCATGCGTCTGCGCGCTCTCAAAGAAACATAGAAGGACGTGAAAAATGGTGGAAA   900
    AAACACTTTCAATTCTCCATTGTCTTGCTAGTGTTTCCTGGGGGCTGTACATATATAAATTATTATTTAAGTTATATAGAATGCATATAG   990
    TTTATACATGTCTATGTATAATGTATAAAGATACTTGTTATTTATTTAAAAGTTATAAAATCTTTGGAAAAAAAA •••             1065
```

(a)

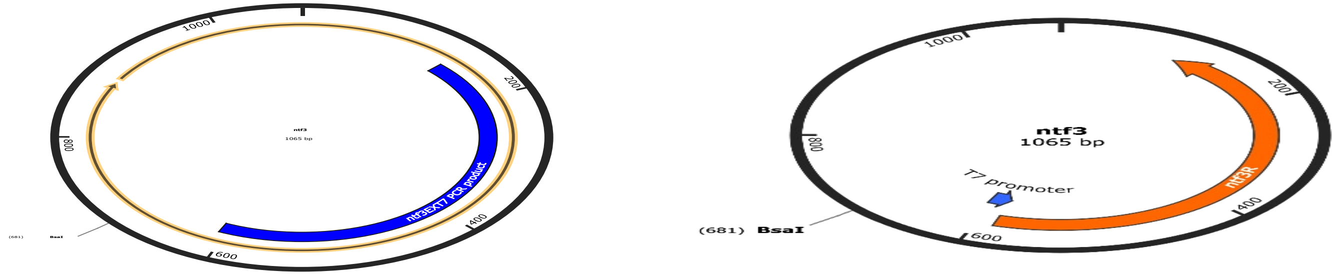

(b) (c)

Figure 1. Amplicon of second PCR using second pair of primer (Table 3.1) gene sequencing. The highlighted codon in A showing the overlap of the codon with the *ntf3* zebrafish mRNA. Figure B shows a schematic representation of the location of the amplicon in comparison to the *ntf3* gene. Figure C shows the amplicon with reverse primer extended with T7 polymerase. This will be the PCR product that will be used for probe preparation.

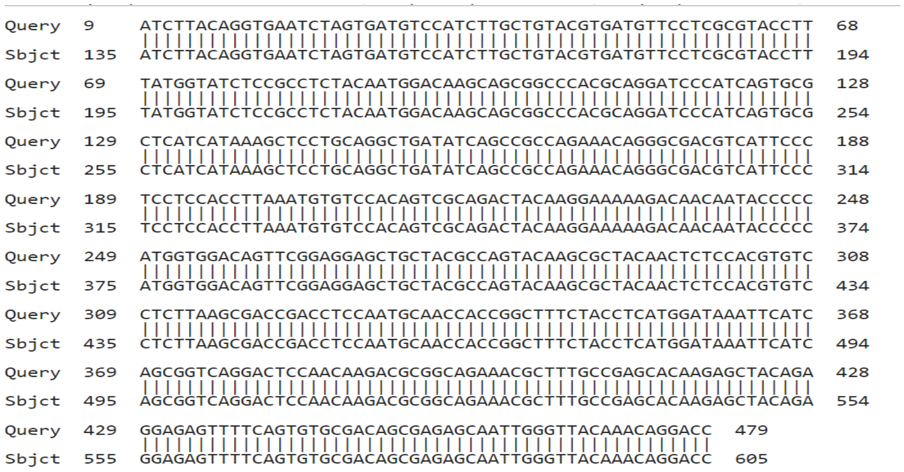

(a)

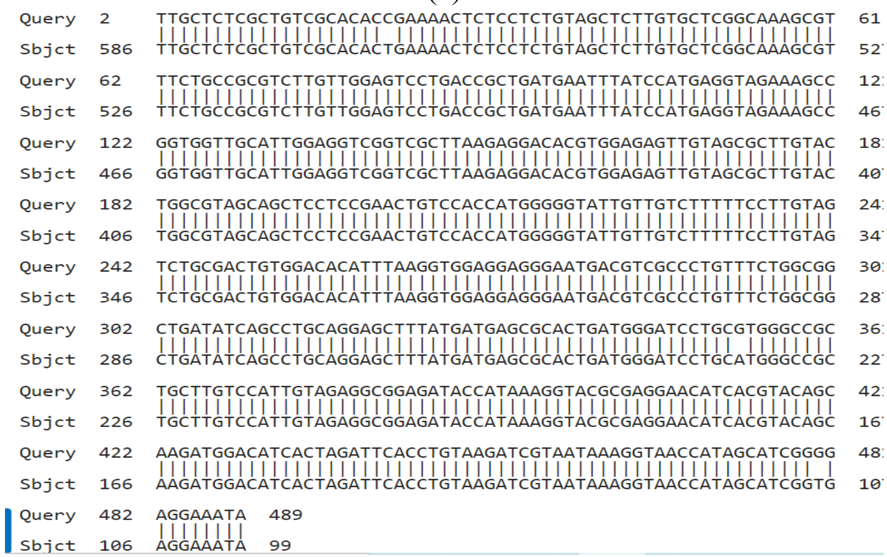

(b)

Figure 4. Mismatched analysis of amplicon of second PCR using the second set of *ntf3* primer (Table 3.1). The query is the amplicon codon arrangement, and the subject is the *ntf3* zebrafish gene acquired from the gene bank, as previously mentioned. The mismatched analysis showed that both forward (A) and reverse primer (B) have a 99% match with the *ntf3* gene of zebrafish.
