# Supplementary material for: Neuroprotective effects of Neurotrophin-3 in MPTP-induced zebrafish Parkinson’s disease model
Source: Front Pharmacol. 2023 Nov 28;14:1307447. doi: 10.3389/fphar.2023.1307447 (PMC10713795; doi:10.3389/fphar.2023.1307447)
Supplement: Supplementary file 2 [file DataSheet3.PDF]

Supplementary 3: Double FIHC images

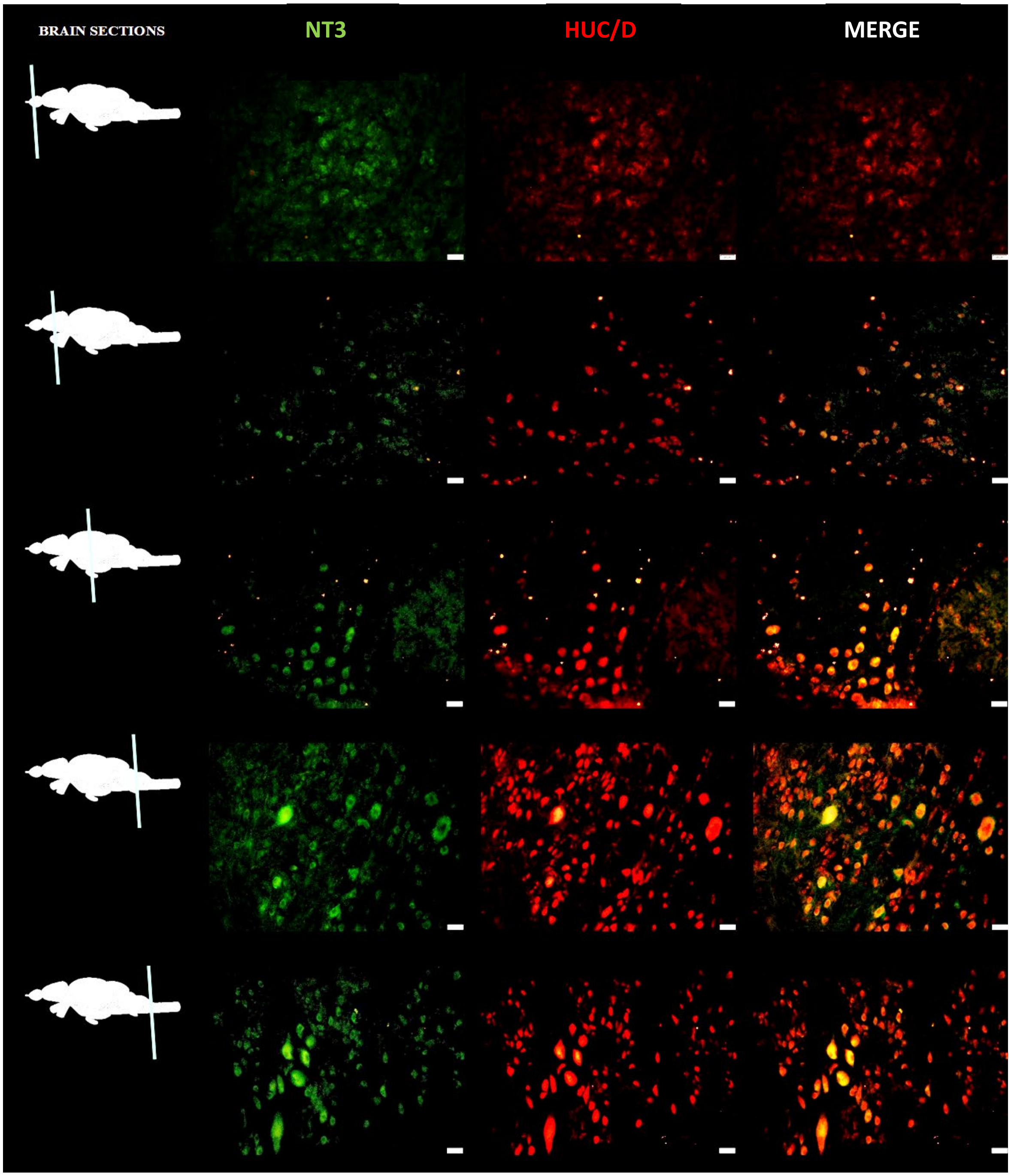

Figure 1. Double FIHC image of NT3 and HUC/D marker showing extensive colocalisation throughout the brain sections. It is observed that all NT3 cells colocalised with the neuronal marker; however, not all neuronal markers express NT3 in their cells. This is especially more prominent in the rhombencephalic area.

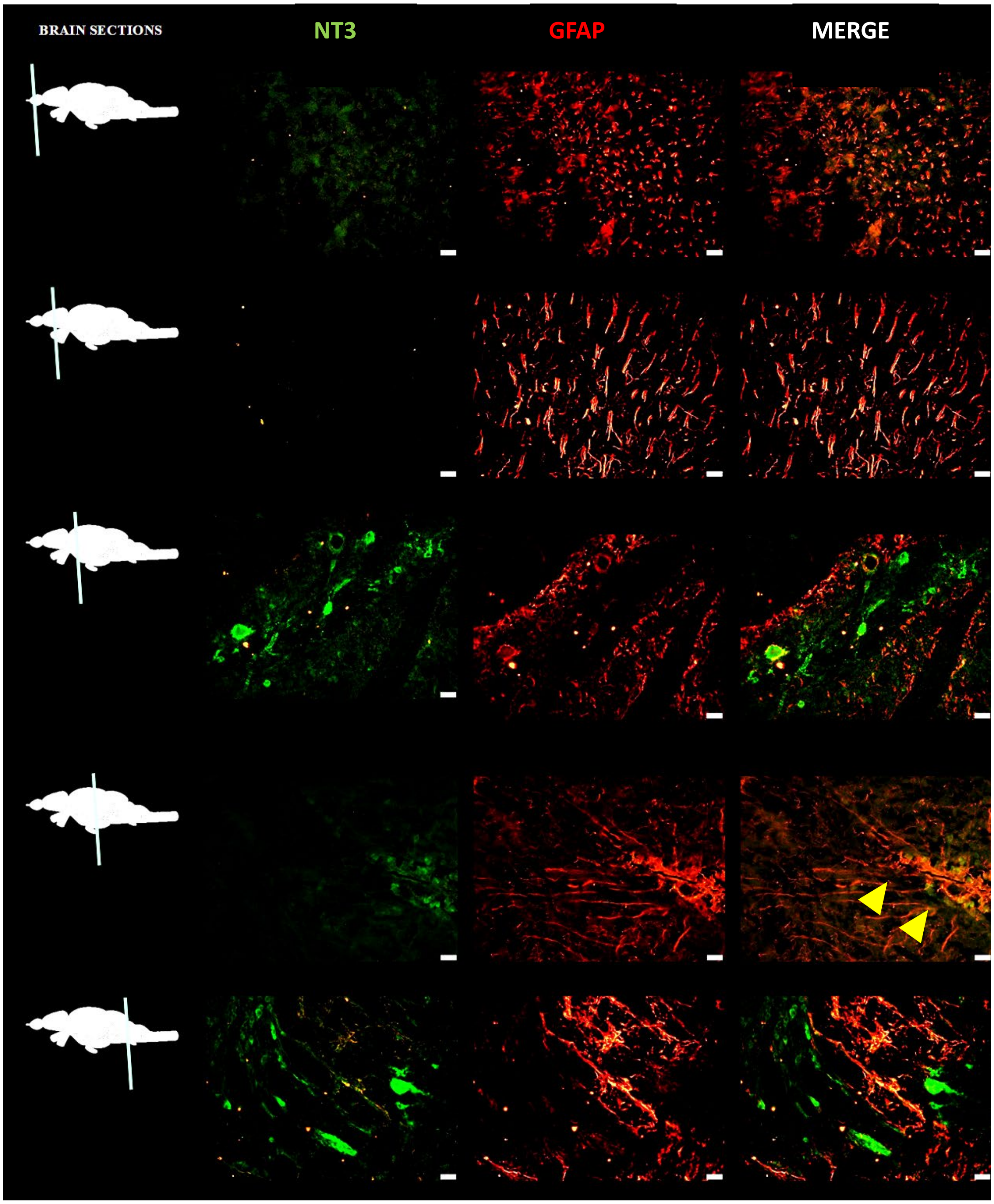

Figure 2. Double FIHC image of NT3 and GFAP marker showing minimal colocalisation throughout the brain sections. It is observed that some clusters of NT3 cells colocalised with the glial marker especially in the diencephalic area (yellow arrowhead).

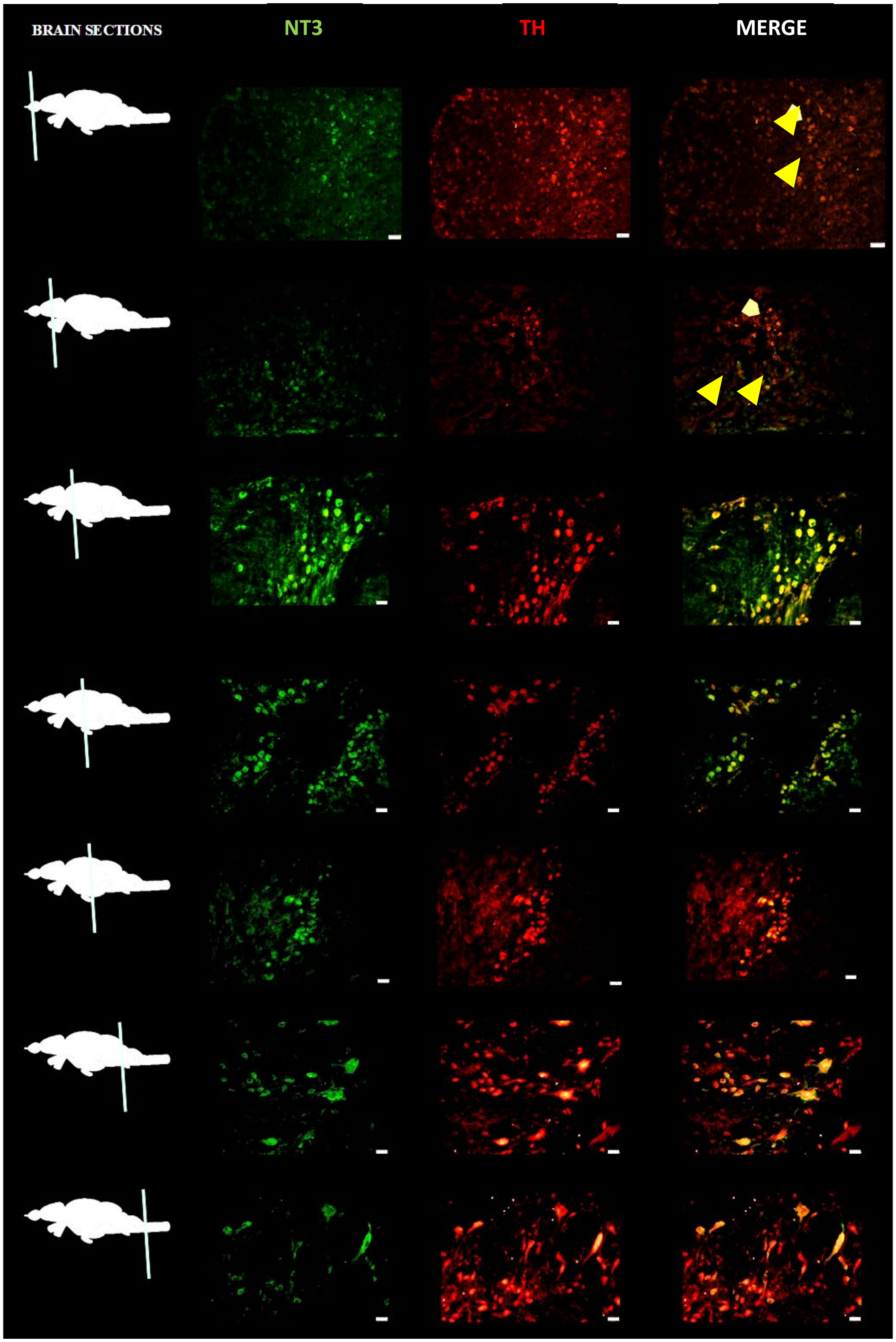

BRAIN SECTIONS
NT3
TH
MERGE

Figure 3. Double FIHC image of NT3 and TH marker showing extensive colocalisation, especially in the diencephalon, mesencephalon surrounding the ventricles, and the rhomboencephalic area. There is minimal colocalisation in the olfactory bulb and the telencephalic area (yellow arrowhead).
