# Supplementary material for: Neuroprotective effects of Neurotrophin-3 in MPTP-induced zebrafish Parkinson’s disease model
Source: Front Pharmacol. 2023 Nov 28;14:1307447. doi: 10.3389/fphar.2023.1307447 (PMC10713795; doi:10.3389/fphar.2023.1307447)
Supplement: Supplementary file 3 [file DataSheet1.PDF]

Supplementary 1: Schematic diagram (methodology)

1. In situ hybridisation

a. Preparation of the probe

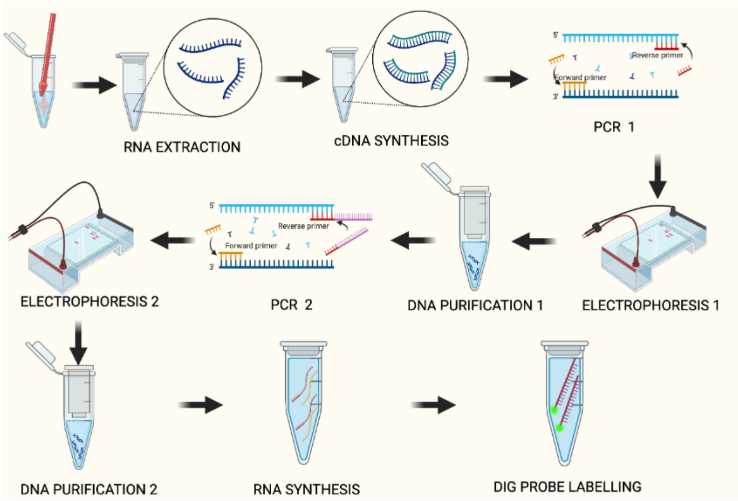

Figure 1. The summary of steps taken to synthesise the *ntf3* DIG-labelled probe. The RNA extracted from the zebrafish brain, underwent reverse transcription to DNA and then multiplication of the *ntf3* DNA using PCR. This was followed by gel electrophoresis and DNA purification. The second PCR step was conducted using T7-extended reverse primer, and the electrophoresis and DNA purification steps were repeated. The purified DNA was converted to RNA and labelled with DIG. The purified DNA was sequenced to ensure accurate gene was synthesised prior to DIG labelling. Obtained probe was used for both chromogenic and fluorescence ISH.

b. Chromogenic ISH

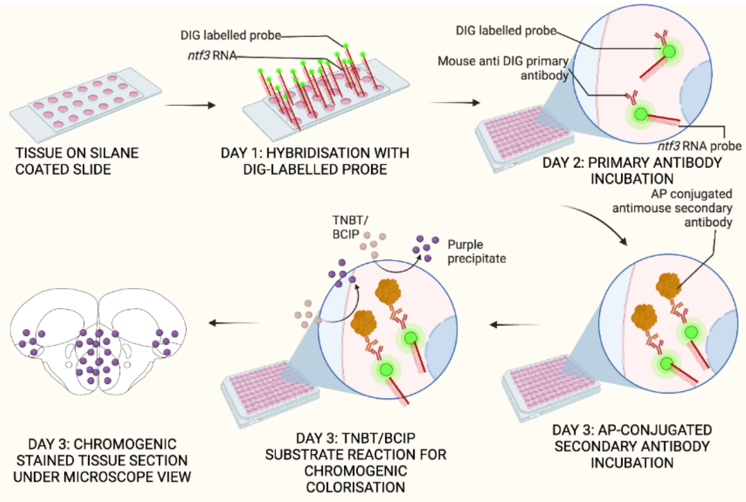

Figure 2. Schematic diagram of the ISH procedure from hybridisation to colourisation. The probe that is incubated on day 1 obtained from the in vitro synthesis in (a).

c. FISH/FIHC

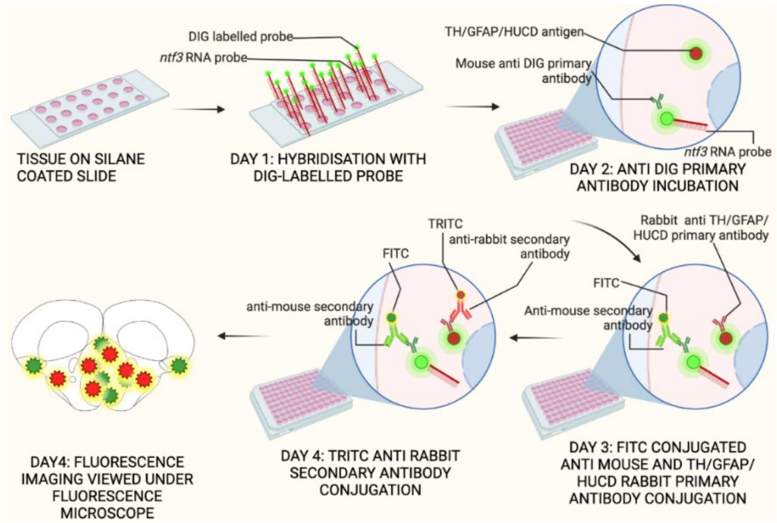

Figure 3. Schematic diagram of double labelling FISH/FIHC procedure. Similar to chromogenic ISH in the initial hybridisation step. However, a second set of antibody incubation was added after the completion of mouse anti-NT3 incubation. The secondary antibody for both antigens was conjugated with fluorescence staining.

2. Immunohistochemistry

a. Chromogenic IHC

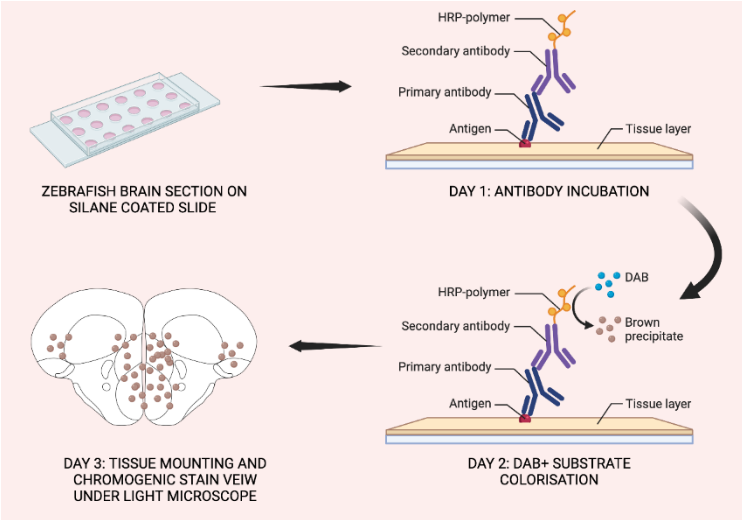

Figure 4. The schematic diagram for chromogenic IHC of zebrafish brain section.

b. Double FIHC

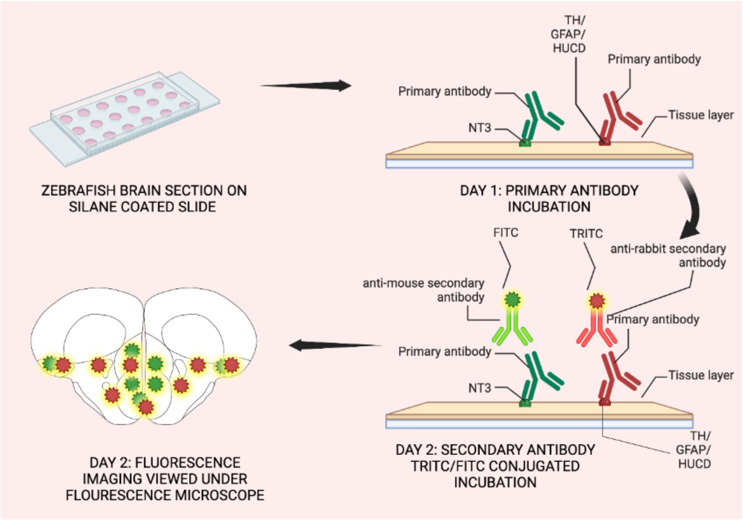

Figure 5. The schematic diagram for double labelling FIHC/FIHC procedure.

Table 1. The list of antibodies that were used in this study.

Marker	Antibody	Host	Dilution	Notes
Neuronal	HuC/D	Rabbit	1:500	Dilution was adapted from a previous study (Yuan et al. 2021).
Glial	GFAP	Rabbit	1:250	Dilution was adapted from previous studies. (Monaco et al. 2016; Pereida-Jaramillo et al. 2021; Shen et al. 2013).
Dopaminergic neuron	TH	Rabbit	1:500	This antibody was used in a double staining procedure with NT3 or *ntf3*.
	TH	Mouse	1:500	This antibody was used in chromogenic IHC staining using the ARK streptavidin kit.
NT3	NT3	Mouse	1:500	Serial dilutions were commenced from 1:200 to 1:1000. The best dilution to ascertain good colourization with minimal background noise is 1:500.
DIG-labelled *ntf3* mRNA	DIG	Mouse	1:200	Adapted from previous study (Qian et al., 2021).
Secondary antibody	Anti-mouse AP-	Goat	1:1000	Adapted from previous studies (Ahsan et al. 2021; Holthaus et al. 2020)

	conjugated			
	FITC anti-mouse	Goat	1:1000	Dilution was adapted from a previous study (Cheng et al. 2015)
	TRITC anti-rabbit	Goat	1:1000	Dilution was adapted from a previous study (Shen et al. 2013)

3. PCR

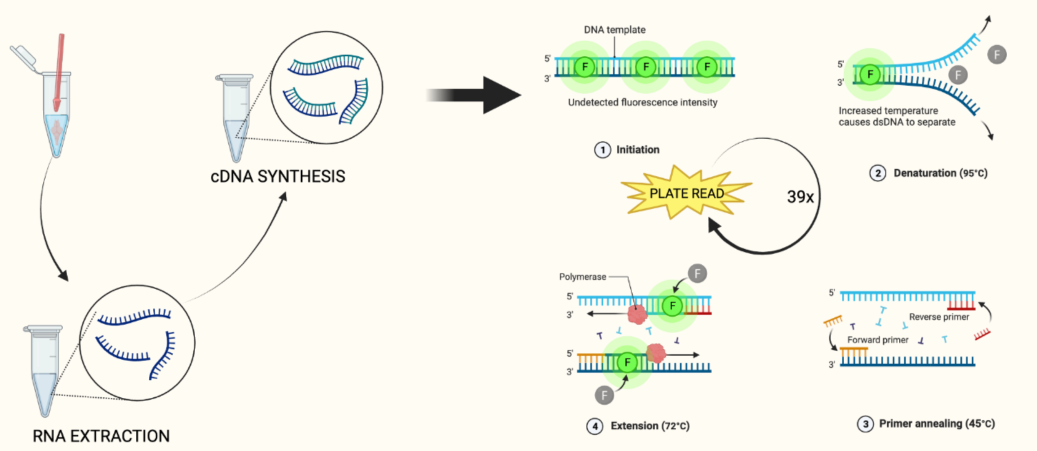

Figure 6. The schematic diagram for the steps required for qPCR.

4. ELISA

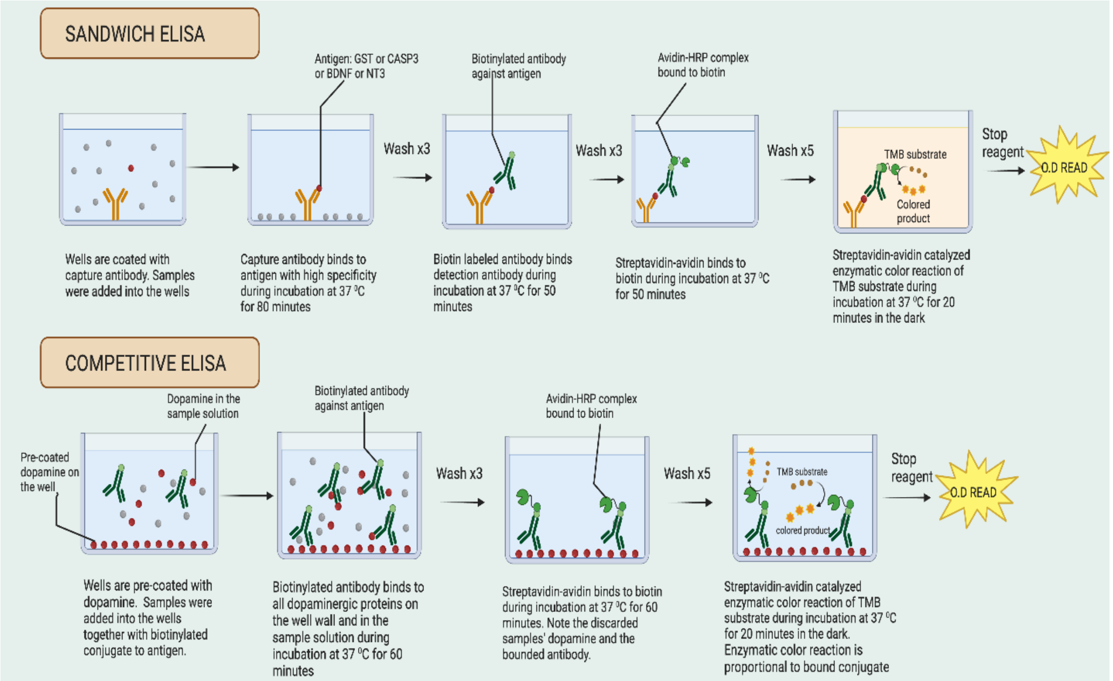

Figure 7. The ELISA tests that were conducted using the zebrafish ELISA kits. GST, CASP3, BDNF and NT3 proteins were assessed using the sandwich method (top), while the DA level was evaluated using the competitive ELISA method (bottom). Note that the sandwich method's detection was based on the bound antibody (on the well) and antigen (protein of interest in the sample solution such as GST, CASP3, BDNF and NT3). Hence OD reading is directly proportional to the protein level in the sample. However, in the competitive ELISA, the wells were coated with the antigen of interest (in this case, the DA). Hence the antigens in the sample compete to bind to their antibody. This was followed by washing the antigen-antibody bound

in the sample solution leaving the antibody-precoated antigen bound on the walls of the wells. The detection of left antibody-antigen bind reflected inversely with the antigen (DA) concentration in the sample since a highly concentrated sample will successfully bind to more free antibodies than the least concentrated sample. Hence the OD level is inversely proportionate to the level of the protein or neuromodulatory molecule tested.
